# Gentle Sterilization of Carrot-Based Purees by High-Pressure Thermal Sterilization and Ohmic Heating and Influence on Food Processing Contaminants and Quality Attributes

**DOI:** 10.3389/fnut.2021.643837

**Published:** 2021-03-18

**Authors:** Maximilian Gratz, Robert Sevenich, Thomas Hoppe, Felix Schottroff, Nevena Vlaskovic, Beverly Belkova, Lucie Chytilova, Maria Filatova, Michal Stupak, Jana Hajslova, Cornelia Rauh, Henry Jaeger

**Affiliations:** ^1^Institute of Food Technology, University of Natural Resources and Life Sciences (BOKU), Vienna, Austria; ^2^Department of Food Biotechnology and Food Process Engineering, Technische Universität Berlin (TU Berlin), Berlin, Germany; ^3^Leibniz Institute for Agriculture Engineering and Bioeconomy (ATB) Potsdam, Berlin, Germany; ^4^Core Facility Food & Bio Processing, University of Natural Resources and Life Sciences, Vienna, Austria; ^5^Department of Food Analysis and Nutrition, University of Chemistry and Technology (VSCHT), Prague, Czechia

**Keywords:** sterilization, high-pressure thermal sterilization, ohmic heating, food processing contaminants, physicochemical properties

## Abstract

Pressure-enhanced sterilization (PES) and ohmic heating (OH) are two emerging sterilization techniques, currently lacking implementation in the food industry. However, both technologies offer significant benefits in terms of spore inactivation using reduced thermal intensity in food products, as well as minimized effects on sensory and nutritional profiles. In this study, PES and OH were tested based on possible food safety process windows in comparison to thermal retorting, to optimize the food quality of carrot-based purees. The following parameters related to food quality were tested: texture, carotenoid content, color, and detectable amount of food processing contaminants (FPC) formed. Application of the innovative sterilization techniques resulted in a better retention of color, texture, and carotenoids (for PES) as well as a reduced formation of food processing contaminants. Importantly, a significant reduction in the formation of furan and its derivates was observed, compared to the retorted samples. Hence, both sterilization technologies showed promising results in the mitigation of potential toxic processing contaminants and retention of quality attributes.

## Introduction

Consumers increasingly demand high-quality foods with a longer shelf life and optimized nutritional attributes. The process currently used in the food industry to achieve a sterile product for low-acid foods is retorting, where applied temperature time profiles can have a negative impact on the nutritional value of the foods (1). Moreover, the formation of processing contaminants, i.e., chemical substances with potentially harmful effects on consumer health, is promoted during severe heat treatments and becomes more and more a subject of scientific and industrial interest (2, 3).

Therefore, novel preservation technologies capable of reducing the thermal process intensity may help to overcome this issue, simultaneously resulting in products with improved sensorial properties with a comparable shelf life yielding a more attractive visual appearance and a better, fresher taste. Among the most promising emerging technologies for the gentle sterilization of foods are, first, high hydrostatic pressure in combination with elevated temperatures, and second, ohmic heating (OH).

For sterilization applications, high pressure (≥600 MPa) can be used in combination with elevated temperatures (90–121°C), as a combined process to inactivate spores (4–9)—the so-called high-pressure thermal sterilization (HPTS) (1, 10, 11). Within HPTS, there are currently two processes that have been accepted by the US Food and Drug Administration (FDA): *First* is the pressure-assisted thermal sterilization (PATS) (12). Here, pressure ≥600 MPa is used for rapid heating purposes only, the synergistic effect of p, T is neglected, only equivalent treatment conditions to thermal retorting are valid, and the PATS process is listed as a thermal process. Second, in 2015, pressure-enhanced sterilization (PES) was accepted by the FDA, where temperatures below 121.1°C at 600 MPa can be applied (13). Here, the synergistic effect of pressure and temperature on spore inactivation was accounted for after intensive research and mechanistic studies to understand the impact of p, T on spores. PES is a possible process window within the pressure (>600 MPa), temperature (110–120°C), and time (1–10 min) domain of HPTS.

The temperature for sterilization can be reached by adiabatic heat of compression when starting temperatures between 70 and 90°C are chosen. Depending on the food system, this temperature increase can range from 3 to 9°C per 100 MPa and represents a rapid volumetric heating that further helps to reduce unwanted thermal effects compared to the effects of conventional heat transfer. The reduction in the thermal effects is due to the synergistic effect of pressure and temperature on the spore inactivation as well as the fact that some thermally driven reactions cannot occur under pressure (1, 14, 15). PES is a promising technology, which has not yet been implemented in the food industry. PES could eventually replace conventional thermal sterilization, producing high-quality, shelf-stable, low-acid foods at lower processing temperatures.

Ohmic heating, on the other hand, is an industrial alternative to traditional heating processes (16–18). For this treatment, alternating current (AC) is directly applied to the food, and heat is generated due to the electrical resistance of the product. In comparison to conventional heating technologies, OH bears the advantages of a volumetric process, i.e., avoiding heat transfer through hot surfaces (19). Additionally, rapid heating at relatively low-temperature gradients inside the product can be achieved if an appropriate configuration of the treatment chamber is chosen. Thus, heating time can be significantly shortened, resulting in a reduced thermal impact and an associated decrease in negative alterations of the product quality at an equivalent sterilization effect compared to conventional thermal processes (20, 21).

Furthermore, PES and OH seemed to have a positive impact on a lower formation of food processing contaminants in food systems, e.g., furan (classified as a potential human carcinogen, 2B) (22), compared to conventional retort sterilization. Baby food formulae are of particular interest in this context, as the presence of processing contaminants in these foods was considered a health concern for toddlers and infants (3). For PES, shorter processing times, Le Chatelier's principle, and lower process temperatures due to additional non-thermal effects may contribute to this phenomenon (23–27). In terms of OH, the volumetric current flow through the product enables the implementation of very rapid heating, leading to improved high-temperature short-time processes with reduced cooking damage (C value) but maintained sterilization value F_0_ (28). Thus, recently, OH was successfully applied to reduce the formation of food processing contaminants during sterilization of vegetable baby food formulations (29, 30).

The mitigation of food processing contaminants, especially furan and acrylamide, is a crucial issue that must be solved by the food industry in the near future because future guidelines will almost certainly include limits for food processing contaminants (FPCs) in foods. Therefore, the aim of this study was to comprehensively assess the benefits of the new processing technologies PES and OH, compared to conventional retort sterilization, considering the effects on product quality and to enable the analytical identification of product quality markers.

## Materials and Methods

In this study, two different volumetric inactivation technologies, i.e., PES and OH, were compared as to the formation of processing contaminants as well as to their influence on product quality characteristics.

### Sample Preparation

Chicken rice puree (CRP) was provided by an industry partner. The recipe was feasible for baby food puree preparation and included the following ingredients: carrots, 40%; water, 33.2%; rice, 10%; raw chicken meat, 8%; turnip cabbage, 4%; onion, 2%, rapeseed oil, 1.5%; and table salt, 0.3%. All samples were taken from the same batch and obtained in a frozen state in 1-kg plastic bags.

Reference (benchmark retorting) samples were processed by a standard industrial retort process (F_0_ = 7 min) and then provided for analysis by the industry partner. Prior to processing, the desired amount of untreated sample was gently defrosted, and the characteristics of the batch were analyzed. Typical values for the CRP were pH = 6.47 ± 0.1, electrical conductivity = 7.35 ± 0.24 ms/cm, water activity = 0.974 ± 0.04, °Brix = 3.75 ± 0.35°, and color values L^*^ = 65.8 ± 0.8, a^*^ = 30.6 ± 2.2, and b^*^ = 60.3 ± 1.6.

Furthermore, to validate and test the analytical method for furan and its derivatives determination [solid-phase microextraction (SPME) gas chromatography–high-resolution mass spectrometry (GC-HRMS) analysis, see *Analysis of Food Processing Contaminants*], two model food systems consisting of carrots, oil, and water (Table 1) were prepared, treated by PES (*PES and Thermal References*) and OH and retort (*Thermal Sterilization by OH and Retort*) and analyzed.

**Table 1:** Recipes of the model food system.

**Recipe puree 1**		**Recipe puree 2**	
Carrots	90%	Carrots	90%
Water	8%	Water	8%
Olive oil (extra virgin)	2%	Rapeseed oil	2%

For this purpose, fresh carrots (*Daucus carota* subsp. *sativus*) were purchased at a local supermarket, peeled, and cut into 1-cm thick slices. Carrot slices were precooked in 90°C tap water for 10 min and subsequently finely pureed (Microcut MC 12, Stephan Machinery GmbH, Hameln, Germany). Subsequently, the carrot puree was mixed with tap water, olive oil, or rapeseed oil, filled in bags, and stored at −30°C until sterilization experiments. To ensure comparability for all used technologies (PES, OH, and retort), the same batch of CRP and of the carrot–oil model food systems were used for the trials.

### Sterilization Treatments

In this study, the effects of high-pressure thermal sterilization (PES) on product quality were compared to those of OH and conventional retort. As OH is considered a predominantly thermal technology (31), the process design was based on the previously validated (data not shown) and well-established concept of F_0_ values (28). It is worth noticing, that an extensive database on inactivation kinetics in a p–T–t landscape for PES is not available to date. Hence, microbial inactivation studies had to be carried out prior to the planned studies on the influence of this treatment on product quality and were used as a basis for later process design. These microbiological analyses are summarized in the Supplementary Material 1 of this paper.

For this study, all trials related to PES were carried out by research partner TU Berlin in Berlin, Germany; OH experiments were accomplished by BOKU in Vienna, Austria; and instrumental analysis was carried out by VSCHT in Prague, Czech Republic. Shipment of samples was carried out in a frozen state by express shipping.

#### PES and Thermal References

**Figure 1:**
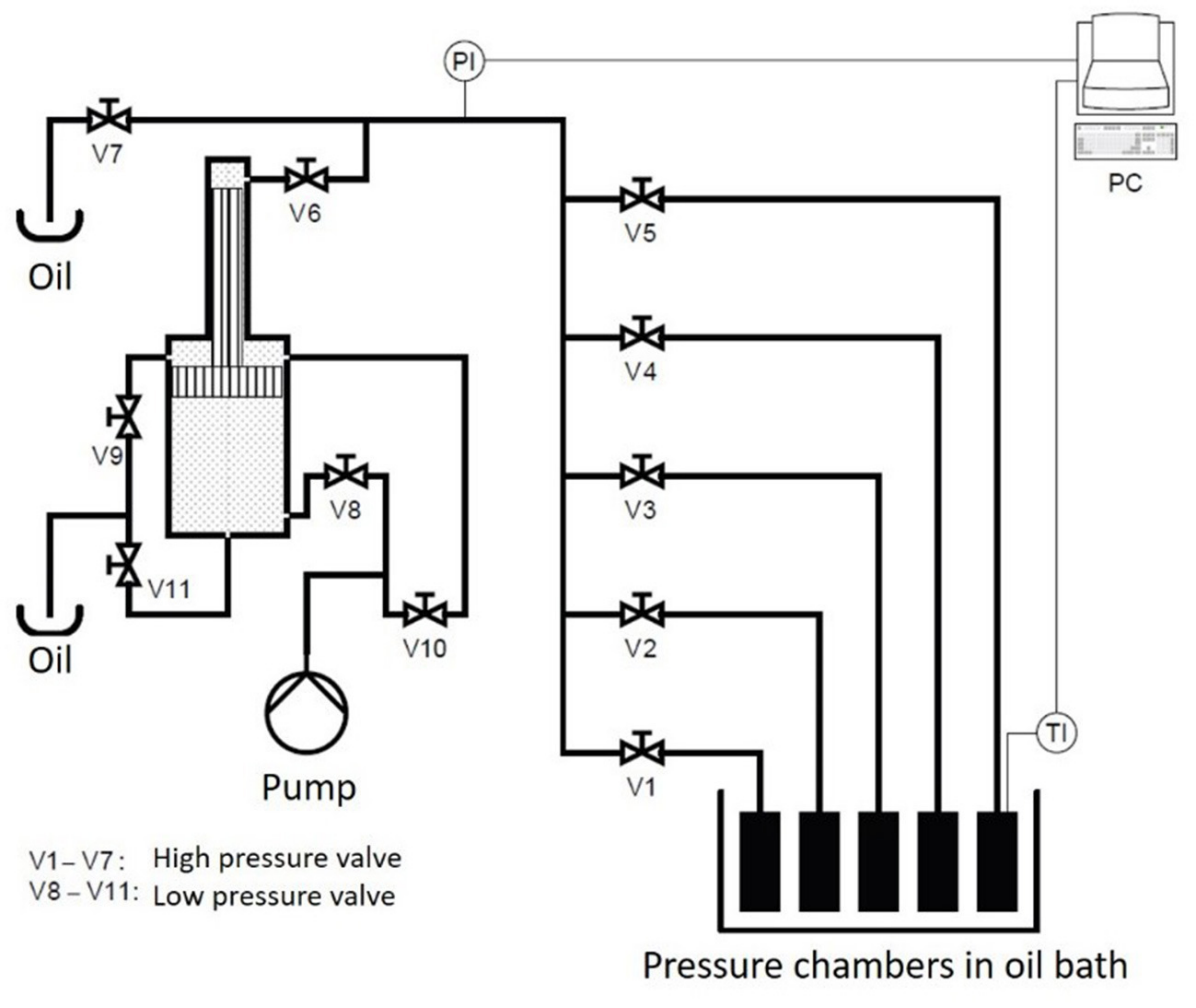
Schematic drawing of the U111 high pressure unit.

The high-pressure unit U111 (Unipress, Warsaw, Poland) was used to conduct the PES studies (Figure 1). With this unit, pressures up to 700 MPa and temperatures up to 130°C can be reached. The high-pressure transmitting medium was silicone oil (M 40.165.10, Huber GmbH, Offenburg, Germany). The unit consists of five chambers, with a volume of 4 ml each, immersed in an oil bath. The pressure build-up rate was 25 MPa/s.

To monitor the temperature during the treatment, a control sample filled with puree was put in one of the chambers equipped with a thermocouple (Unipress, Warsaw, Poland) placed in the geometrical center of the sample. The temperatures selected for the treatment were between 105 and 121°C at 600 MPa with treatment times from 0.166 up to 100 min. The oil bath of the U111 was set to the selected process temperature plus 5°C, and the start temperatures for the food system were obtained in pretrials (Table 2).

**Table 2:** Empirical values for the start of high-pressure treatment of the chicken rice puree (CRP).

**T_**oil**_ (°C)**	**T_**ch**_ (°C)**	**T_**st**_ (°C)**	**T_**end**_ (°C)**
109	106	60	102
111	108	62	104
113	110	64	106
115	112	66	108
117	114	68	110
119	116	70	112
121	118	72	114
123	120	74	116
125	122	76	118
127	124	78	120

*T_oil, temperature of the oil bath; T_ch, temperature in the sample chamber; T_st, temperature at the start of the pressure build-up; T_end, target treatment temperature of the sample*.

The trials for the analysis of food processing contaminants and quality parameters were completed in duplicates with samples of the model food purees or the CRP. Portions of the mixed matrix (1.5 g) were placed in a container (Nunc Cryo Tubes Nr. 375299, Nunc A/S, Roskilde, DK) and put into an ice bath. After the treatment, the samples were put immediately on ice to reduce further formation of processing contaminants and then frozen at −80°C. The carrot–oil model food system was treated at different temperatures (105, 110, and 115°C) for 5–10 min with and without pressure to observe furan formation.

The thermal references were conducted in the same container (1.5 g) using the same T, t combinations (105, 110, and 115°C for 5–10 min at 0.1 MPa) as the PES-treated samples. A thermostatic bath (cc2, Huber GmbH, Offenburg, Germany) filled with silicon oil (M40.165.10, Huber GmbH) was used for the thermal reference treatment. Following thermal treatment, the samples were immediately transferred to an ice bath. The selected treatment conditions are shown in Table 3. For the CRP, the aim was to find p, T, t combinations that provided a 12 log_10_ inactivation of *Bacillus amyloliquefaciens* to ensure a safe sterilization treatment. To find suitable process windows, inactivation trials were done as described in the Supplementary Material 1. The resulting process conditions are shown in Table 3.

**Table 3:** Process conditions for carrot–oil model food systems and chicken rice puree (CRP) treated by pressure-enhanced sterilization (PES) (600 MPa) and thermal references (0.1 MPa) with the same temperature history.

**Sample**	**Parameters PES and thermal reference**	**Sample**	**Parameters PES**
Carrot–oil model food puree	600 MPa and/or 105°C, 5 min	CRP	600 MPa, 110°C, 9.9 min
	600 MPa and/or 105°C, 10 min		600 MPa, 112°C, 7.9 min
	600 MPa and/or 110°C, 5 min		600 MPa, 114°C, 6.5 min
	600 MPa and/or 110°C, 10 min		600 MPa, 116°C, 5.2 min
	600 MPa and/or 115°C, 5 min		600 MPa, 121°C, 7.0 min
	600 MPa and/or 115°C, 10 min		

In the food industry, F_0_ values (referring to 121°C reference temperature and a z value of 10°C) between 4 and 10 min are commonly used for thermal sterilization treatments (32). However, a comparison with high-pressure sterilization in terms of spore inactivation is difficult because at high temperature and 600 MPa, the inactivation can be very sudden and rapid due to additional, non-thermal effects (9). Therefore, only the C value concept was applicable to compare the cooking damage between retorting, OH, and PES (see *C Value Calculation*).

#### Ohmic Heating

OH was performed using a pilot-scale generator (German Institute of Food Technologies, DIL, Quakenbrück, Germany). The generator applied rectangular bipolar pulses at a pulse repetition rate of 12 kHz. The system possesses a maximum power of 15 kW with a maximum voltage of 500 V (peak). By adjustment of the pulse width (between 10 and 40 μs), a constant power was applied during the treatments.

**Figure 2:**
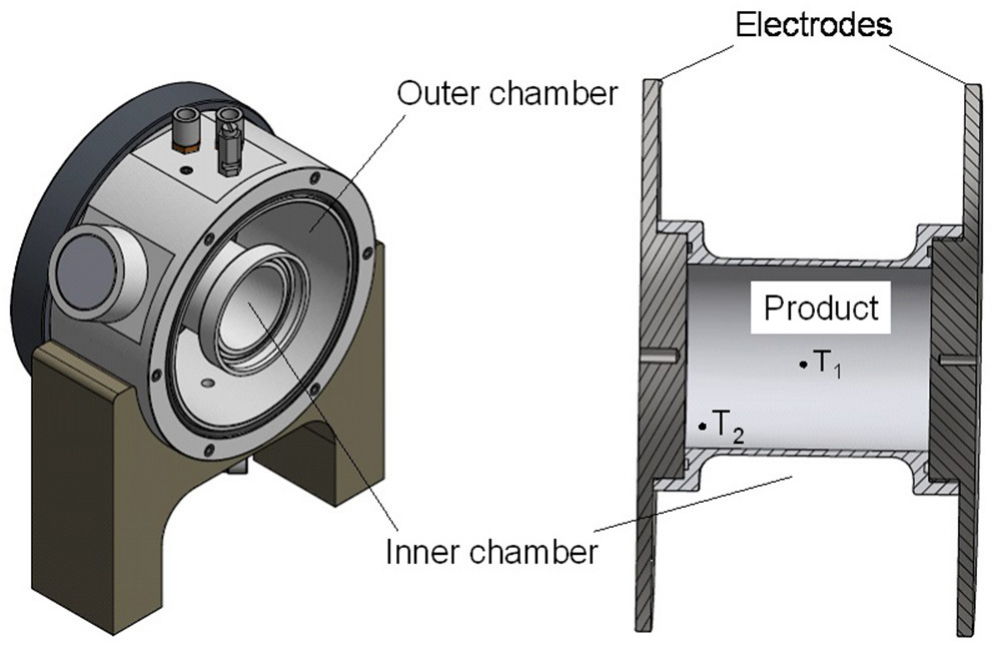
Drawing of the ohmic heating (OH) treatment cell for sterilization applications, including the double jacket cell (left) showing the outer and inner chamber and the inner treatment chamber (right), with the positions of the temperature sensors (T_1_ and T_2_).

The trials were carried out using a self-built cylindrical double jacket chamber designed to provide uniform heating profiles at sterilization conditions. The body of the chamber was made of polyether ether ketone (PEEK), and the electrodes consisted of stainless steel (Figure 2). The chamber was built to allow runs at temperatures above 130°C and to minimize cold spots on the outside by simultaneously heating the double jacket (outer chamber) during OH.

The inner chamber (230 ml) for the treatment of the product was designed to geometrically and volumetrically resemble the glass jars used for retort sterilization, with a diameter of 60 mm and a length of 80 mm. Together with the outer chamber, the total filling volume was 1.8 L. For the sterilization runs, 2 kW power was applied. Purees were filled into the chamber at room temperature and heated by the application of the electric field until the desired F_0_ value was reached in the inner chamber (at the coldest spot). The outer chamber was filled with salt water and heated by application of OH as well. Cooling was provided by flushing the outer chamber with cold water. When the core temperature (T_1_) was below 50°C, samples were removed, quickly put into sample containers, and immediately stored at −30°C.

Temperature was monitored using four polytetrafluoroethylene (PTFE)-coated K-type thermocouples (Ellab GmbH, Gyhum, Germany), two in the inner product chamber and two in the outer chamber. The sensors in the inner chamber were placed in the geometrical center (T_1_) and close to the electrode in the lower corner of the chamber (T_2_). Based on preliminary trials, position T_2_ was chosen as a potential cold spot during OH, due to both the conductive heat loss toward the electrode and the convective heat transfer toward the top of the chamber.

#### Thermal Sterilization by OH and Retort

Although some data from the literature suggest that sterilization by OH could result in an additional non-thermal inactivation of bacterial spores (33–35), data are still scarce on this topic. In our pretrials (data not shown), no additional inactivation by OH in carrot puree was observed. Therefore, in this study, the sole thermal sterilization effect was considered to ensure a safe and comparable OH sterilization process. For purposes of comparison, OH and the conventional retort sterilization processes were performed at the same F_0_ values. The common C value was determined as an additional indicator for the thermal load affecting the quality characteristics.

For the conventional retort sterilization, the carrot–oil model purees were preheated to 80°C and filled into glass jars (230 ml), which were then sterilized in a static retort system (Type YRX 900-0 BV, dft technology GmbH, Neumünster, Germany) by spraying hot water (121°C) on the jars. Retorted CRP samples (benchmark), provided by the industrial partner for further analysis, were also sterilized in glass jars (250 ml).

For the OH sterilization runs, the vegetable puree was deposited into the inner treatment chamber (Figure 2) at room temperature. The outer chamber was filled with salt water; the conductivity matched to the product. During the heating process, the salt water in the outer chamber and the product in the inner chamber were heated simultaneously to prevent cold spots close to the wall and on the contact surfaces with the electrode. As similarly reported by Zell et al. (36), a reduction in cold spots by external heating was observed, in this case by the simultaneous heating of salt water in the outer chamber.

To achieve a uniform overall thermal load, the process had to be designed in such a way that slightly higher heating rates resulted in spots close to the wall of the inner chamber. This was necessary, as the subsequent cooling step (flushing the outer chamber with cold water) cooled the volume elements of the sample close to the cylinder wall more quickly due to conventional heat transfer. An increased heating rate in the outer chamber also increased the heating rates in those areas of the inner chamber, thus achieving similar lethality values in the center (T_1_) and close to the cylinder wall and the electrode in the lower corner (T_2_) (Figure 2). The increased heating rate of the outer chamber was achieved by increasing the conductivity of the surrounding water.

The carrot puree (Table 1) was sterilized by OH and retorted at four different F_0_ values (F_0_ = 3, 7, 14, and 21 min), covering a range of F_0_ values used in industrial sterilization treatments.

To ensure the comparability of results, the CRP was sterilized by OH at the same F_0_ value (7 min) as the industrial benchmark retort sample. For the ohmic treatments, the maximum temperature varied while maintaining a constant F_0_ value. Thus, CRP was sterilized at maximum temperatures of 115, 121, 125, and 130°C. In the tests, the rapid heating rates resulting from OH were used to quickly reach the desired temperature with the intention of reducing the thermal load. Finally, it was tested which parameters would lead to better preservation of valuable ingredients or to a minimization of processing contaminant formation: a lengthy treatment at reduced temperatures or an abbreviated treatment at higher temperatures. All runs were performed in duplicates.

#### C Value Calculation

To be able to compare the impact of the different sterilization methods on the physiochemical properties and furan content at a comparable level of thermal damage, cooking values (C values) were determined for all treatments. The C values (min) were calculated from the temperatures measured in the core of the samples (T_1_) according to Equation 1 (37).

(1) $$\begin{array}{l} C=\int_{0}^{t}10^{\left(\frac{T-T_{ref}}{z}\right)}dt \end{array}$$

with t = treatment time (min), T = temperature (°C), T_ref_ = reference temperature (set to 100°C) and z = temperature change that induces a 10-fold change of the D value. A z value of 30°C was used, as similar values were used in the literature for changes in the physiochemical properties (38).

### Analytical Determination of Product Quality

#### Color

All color measurements were carried out using an image analysis system (DigiEye color measurement and imaging system, Verivide Limited, Leicester, UK). The CRP samples were spread uniformly in the bottom of a Petri dish and placed in the middle of a photo box to guarantee reproducible light conditions. Photos were taken using a digital camera (D90, Nikon Corp., Tokyo, Japan). Based on the images, L^*^, a^*^, and b^*^ values were determined using the integrated software (Digipix, Verivide Limited, Leicester, UK).

From the obtained L^*^, a^*^, and b^*^ values, ΔE values were calculated in reference to the untreated sample for the retort benchmark samples, ohmically heated samples, and PES-treated samples (Equation 2). This value indicates the overall difference between two colors. According to the literature, for ΔE values above 2.3, a slightly noticeable color difference can be perceived (39).

(2) $$\begin{array}{l} \Delta E_{S,R}=\sqrt{{\left(L_{S}^{*}-L_{R}^{*}\right)}^{2}+{\left(a_{S}^{*}-a_{R}^{*}\right)}^{2}+{\left(b_{S}^{*}-b_{R}^{*}\right)}^{2}} \end{array}$$

with L^*^, a^*^, and b^*^ values determined from a certain sample (S) compared to a defined reference (R)—in this case, the untreated CRP sample.

#### Texture

Textural profile analyses (TPAs) of the PES-treated CRP samples were conducted using a texture analyzer (1 kN Zwicki, MPMS 50302, Zwick GmbH & Co. KG, Ulm, Germany) according to Shim and Lim (40). Texture parameters such as firmness, adhesiveness, cohesiveness, springiness, and gumminess were measured in triplicate. The following instrumental test parameters were used: mode was forced in compression; load cell value was 1 kN; trigger type was 0.1 N; and a cylindrical plastic probe (25 mm diameter, 40 mm height) was used. An aliquot of each sample (~100 g) was analyzed in a plastic sample beaker (50 mm diameter, 105 mm height) resulting in a sample height of around 50 mm. The entry depth of the probe was set to 10 mm using a test speed of 50 mm/min. Samples were analyzed at room temperature. To determine the texture of the samples, the surface layer of each sample was smoothened, and the test cycle was started recording the force–time curve. The force–time curve was further evaluated to determine the texture parameters using an octave code that had been programmed in-house according to the interpretation rules for textural profile analysis.

A TPA of both the ohmically and the benchmark retort sterilized samples were done with the same parameters as for the PES samples using a Stable Microsystems texture analyzer (Texture Analyzer XTplus, Stable Microsystems Ltd, Godalming, England). The force data were recorded, and TPA parameters (i.e., firmness, adhesiveness, cohesiveness, springiness, and gumminess) were calculated by the Exponent Texture analyzer software (Stable Micro Systems Ltd.).

#### Analysis of Food Processing Contaminants

##### Furans

Head space solid-phase microextraction (HS-SPME) with gas chromatography coupled to high-resolution time of flight mass spectrometer Pegasus HRT (LECO, USA) was used for the determination of furan and its derivates (2-methylfuran, 3-methylfuran, 2,5-methyfuran). Sample preparation and measurement were performed using a slightly modified method by Hradecky et al. (30). The analytical approach represents a certified accredited method according to EN ISO/IEC 17025:2018.

##### Acrylamide

Ultrahigh-performance liquid chromatography using an Acquity liquid chromatograph system (Waters, USA) coupled to a XEVO TQ-S Tandem Quadrupole Mass Spectrometer (Waters, USA) was used for detection and quantification of acrylamide. Analytical approach was conducted as described by Forstova et al. (41). The analytical approach represents a certified accredited method according to EN ISO/IEC 17025:2018.

##### 3-MCPD Esters

Ultrahigh-performance liquid chromatography (U-HPLC) using an Acquity Ultra high-pressure liquid chromatograph system (Waters, USA) coupled to high-resolution orbitrap mass spectrometer Exactive™ (Thermo Fisher Scientific, Bremen, Germany) was used for the determination and quantification of individual 3-monochloropropane-1,2-diol (3-MCPD) bound in diesters (expressed as 3-MCPD esters). Sample preparation and measurement were performed with a slight modification described by Moravcova et al. (42). The analytical approach represents a certified accredited method according to EN ISO/IEC 17025:2018.

#### Analysis of Carotenoids

HPLC coupled to diode array detector (DAD) was used for the determination of carotenoids. The analytical approach was performed as described by Bhave et al. (43) with a slight modification including alkaline hydrolysis prior to carotenoid extraction. Briefly, 2 ml of a mixture of ethanol/acetone (6:4, *v*/*v*) with 0.2% (*w*/*w*) of tert-butyl-hydroxytoluene (t-BHT) was added to 1 g of sample. The mixture was vigorously shaken. Afterwards, 6 ml of hexane followed by 5 ml of the ethanol/acetone mixture was added and shaken for 5 min. A solution of KOH (5 mL) was added and left for 18 h.

The samples were neutralized with Na_2_SO_4_ after hydrolysis, and another 10 ml of hexane was added, shaken for 2 min, and centrifuged at 10,000 g for 5 min. The extraction of carotenoids was repeated with 10 ml of hexane, altogether three times. The hexane layers were collected into an evaporating flask and evaporated to dryness on a vacuum rotary evaporator. The residue was then reconstituted in 4 ml of ethanol/acetone (6:4, *v*/*v*) with 0.2% (*w*/*w*) of t-BHT and microfiltered by 0.2 μm polyvinylidene fluoride (PVDF) membrane filter (43).

## Results and Discussion

### Evaluation of Different Sterilization Processes

All three technologies, PES, OH, and conventional retort, were successfully applied to sterilize the carrot–oil model purees and the CRP. OH and traditional retort samples were sterilized by the same F_0_ values, carrot–oil purees between F_0_ = 3–21 min, and CRP at F_0_ = 7 min. PES samples were sterilized by p, T, t combinations that equaled a 12 log_10_ inactivation of *B. amyloliquefaciens* (Supplementary Material 2.1). The calculated C values based on the core temperatures of the samples during the treatments were used solely to estimate the thermal impact of the treatments with regards to the cooking damage and to compare the obtained results.

**Figure 3:**
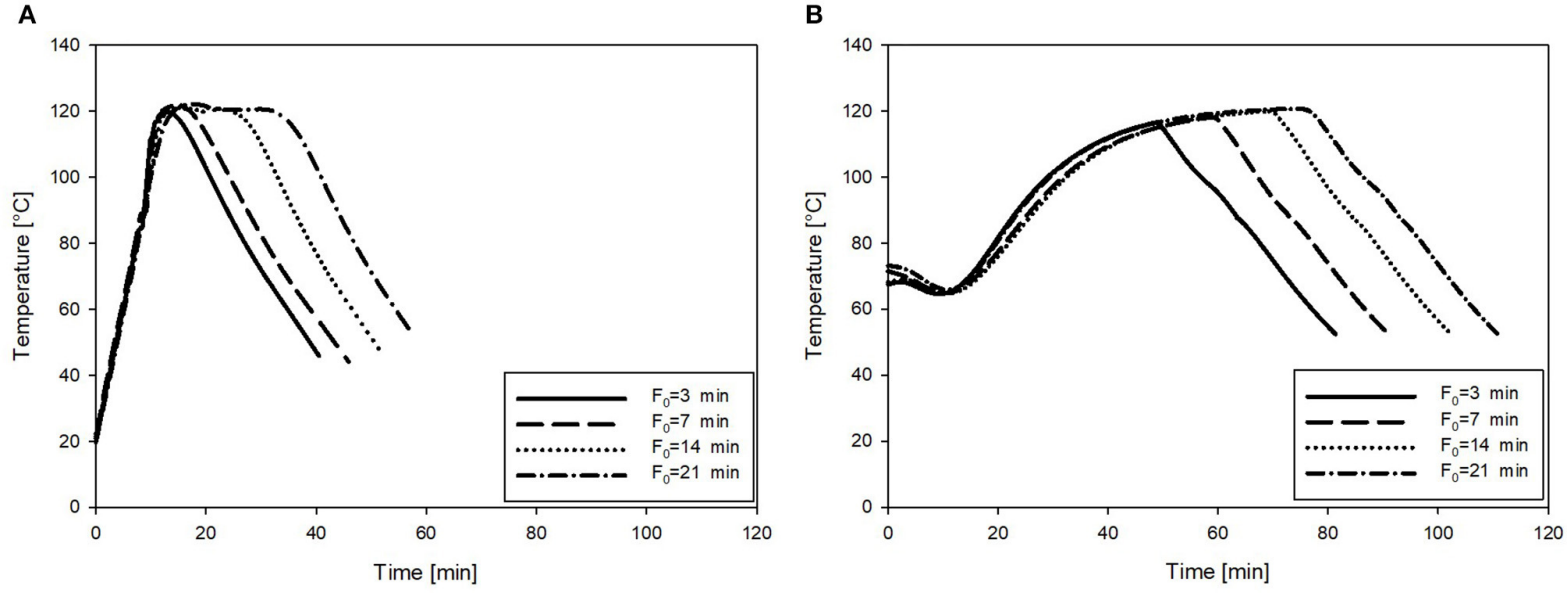
Exemplary temperature profiles of the carrot–oil puree sterilized at F_0_ = 3, 7, 14, and 21 min. **(A)** Treated by OH and **(B)** heated by the conventional retort.

The retort sterilization resulted in a slow heating rate, demonstrating the necessity of preheating of the product to 80–85°C prior to sterilization. These conditions represent common practice in the industry. On the other hand, OH treatments resulted in a more rapid heating of the puree samples, rendering the separate preheating step obsolete. To illustrate this, the core temperature profiles during the retort and OH sterilization (F_0_ = 3–21 min) of the carrot–oil model medium are shown in Figure 3.

Additionally, the time needed to reach the target F_0_ values and the resulting C values of all treatment combinations are summarized in Table 4. As expected, both the treatment time and the C values increased with increasing F_0_ values in the carrot–oil model medium treated with both technologies. However, for OH, treatment time and resulting C values could be significantly reduced at all four F_0_ values compared with those of the retort treatment. These results can be linked to the more rapid and uniform heating profiles observed during the OH treatment. The reduction in treatment time and C value relative to the retort treatment was higher for the lower F_0_ values. At F_0_ = 3 min and 7 min, the reduction in the time to reach the respective F_0_ value was over 60%; at F_0_ = 21 min, it was 51%. For the C value, the reduction by OH treatment compared to the retort treatment was 41% at F_0_ = 3 min, while at F_0_ = 21 min, the reduction was only 21%. Those observations were expected for higher F_0_ values (also for OH treatment); thus, longer holding times at 121°C were necessary, which contributed to enhanced treatment times and C values. To better benefit from the OH treatment for the higher F_0_ values, higher maximum temperatures (T_max_) could considerably shorten the temperature holding time, therefore further enhancing the thermal load reduction relative to the retort treatment.

**Table 4:** Time to reach the target F_0_ value and resulting C values of carrot–oil model medium sterilized by ohmic heating (OH) or conventional retort.

	**Time (min)**		**C value (min)**	
**F_**0**_ value (min)**	**OH**	**Retort**	**OH**	**Retort**
3	19.2 ± 5.3	49.6 ± 3.6	43.3 ± 4.1	73.3 ± 16.4
7	23.5 ± 3.7	63.7 ± 2.2	67.0 ± 6.4	104.5 ± 12.4
14	31.2 ± 2.4	73.4 ± 0.9	100.7 ± 5.3	145.0 ± 8.3
21	40.1 ± 7.1	82.8 ± 7.1	150.4 ± 15	190.0 ± 12.6

**Figure 4:**
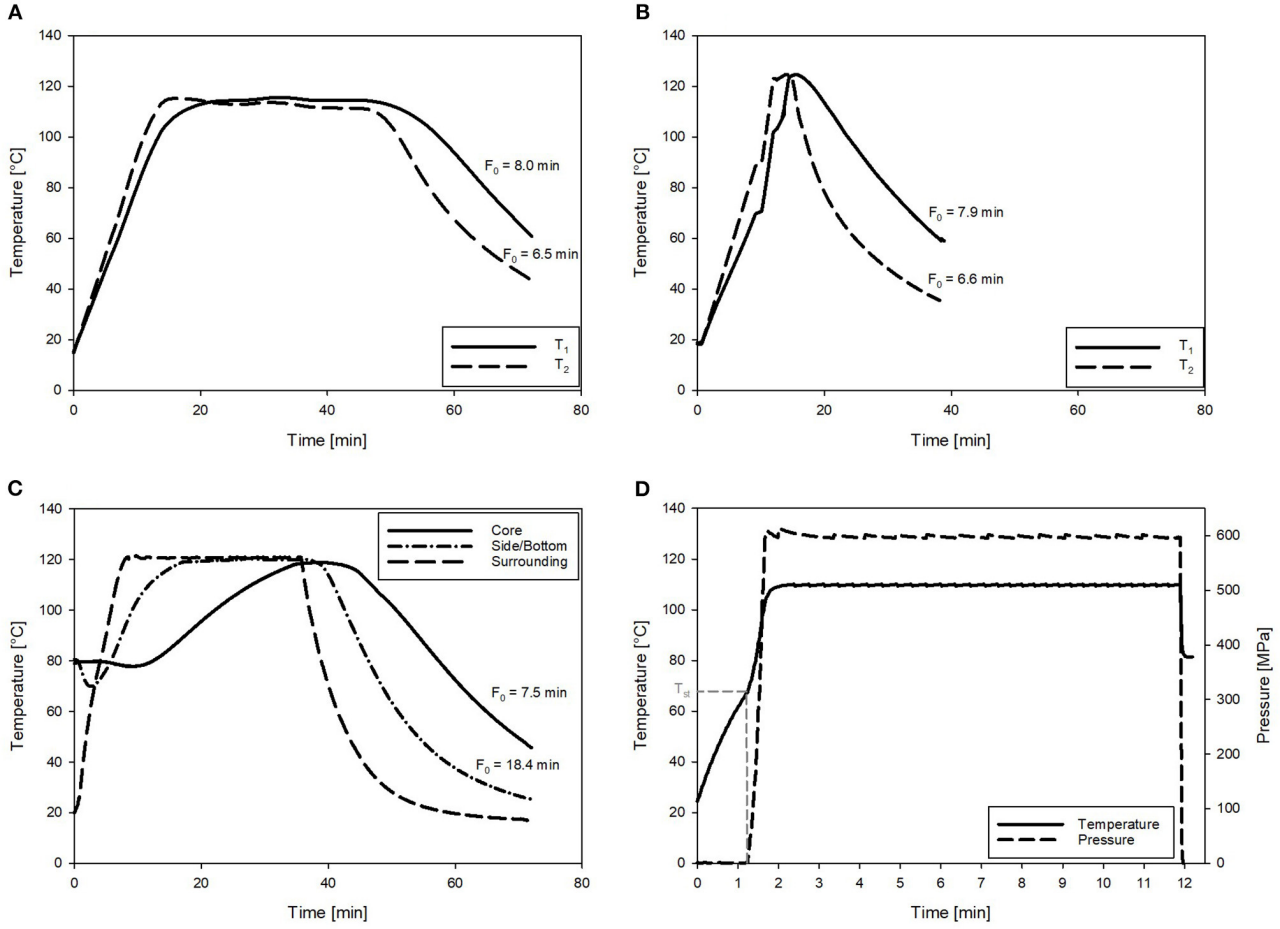
Exemplary temperature profiles and corresponding F_0_ values of the chicken rice puree (CRP) sterilized by ohmic heating (OH), retort, and pressure-enhanced sterilization (PES). **(A)** OH with T_max_ = 115°C. **(B)** OH with T_max_ = 125°C (T_1_ and T_2_ are referring to temperature sensor positions in the core and close to the electrode as defined in Figure 2). **(C)** Retort sterilization with one sensor in the geometrical center and one in the lower corner of the glass jar. **(D)** PES sterilization at 600 MPa, 110°C for 9.89 min. With the pressure–temperature curve and T_st_ referring to the start temperature for pressure build-up.

Therefore, to reduce the overall thermal load for the CRP samples, varying T_max_ for the OH treatments were investigated. As expected, with higher T_max_ for the OH treatments, time period to reach a F_0_ = 7 min and C values decreased (Table 5). However, this was only observed until a T_max_ of 125°C. Due to the slow cooling of the samples with a T_max_ of 130°C, F_0_ values were above F_0_ = 7 min, and therefore, the C values were also higher compared to T_max_ of 125°C. Compared to industrial benchmark retort sterilization, the time period needed to reach F_0_ = 7 min could be reduced by a maximum of 71%, including a reduction of 57% for the attained C value, which was determined for the OH treatment with a T_max_ of 125°C. Furthermore, in terms of ΔT within the samples during heating, the OH treatment exhibited a clearly superior heating uniformity. Benchmark treatment resulted in an over-processing of the volume elements on the outside and lead also to higher F_0_ and C values in these areas. Similar results could be attained for F_0_ and C values using OH (Figure 4).

**Table 5:** C values and actually reached F_0_ values of chicken rice puree (CRP) sterilized by ohmic heating (OH), industrial benchmark retort, and pressure-enhanced sterilization (PES).

**Parameters**	**C value (min)**	**F_**0**_ (min)**	**Parameters**	**C value (min)**
Benchmark	98.7 ± 5.4	7.4 ± 0.2	PES 110°C, 9.89 min	20.0
OH T_max_ 115°C	125.4 ± 2.1	7.5 ± 0.1	PES 112°C, 7.9 min	16.9
OH T_max_ 121°C	70.1 ± 7.2	6.9 ± 0.7	PES 116°C, 5.22 min	13.5
OH T_max_ 125°C	45.1 ± 8.6	7.4 ± 3.5	PES 121°C, 7 min	41.4
OH T_max_ 130°C	72.5 ± 8.9	13.2 ± 1.4	Thermal 121°C, 7 min	65.3

*PES parameter combinations provided at least a 12 log_10_ inactivation of Bacillus amyloliquefaciens*.

A more rapid and efficient heating of vegetable and fruit purees by batch OH was also reported by several other authors (44, 45). Yildiz et al. (46) reported up to 400% decrease in the heating time period required for spinach puree by OH (up to 40 V/cm) as compared to that of conventional heating. The same working group also investigated the blanching of pea puree by OH and found similar results, namely, that OH could reduce the necessary blanching time sixfold (47).

The PES sterilization treatments resulted in even lower C values (Table 5). The synergistic inactivation effect of the applied pressure and temperature allowed for a reduction in both the processing temperature and the thermal load, compared to those of the retorted carrot–oil puree and the industrial benchmark CRP samples (Supplementary Material 2.1). For the CRP, it is evident that the thermal load applied to the product, depending on the process (except for PES 121°C, 7 min), was 69–79% lower for the industrial benchmark process. Even for treatments with comparable holding times of 7 min at 121°C, the PES sample and the thermal reference sample exhibited differing C values. This was due to different T, t profiles corresponding to variable heating and cooling patterns. The thermal load of the PES process was 36% lower in comparison to that of the industrial benchmark process. The selected parameters from Table 5 are the basis of all other conducted trials.

Hence, the results show that with both volumetric sterilization technologies (OH and PES), a reduction in the overall thermal load through a more uniform and efficient heating mode was possible. Both technologies were able to provide distinctly higher heating rates and possess, therefore, the potential fora radical reduction in process time requirements.

In scientific literature, additional non-thermal microbial inactivation effects attributed to OH were discussed (33, 35, 48). However, until now, the available data are contradictory. Potential additional inactivation mechanisms caused by the electric field during OH were not considered in this study. The reduction in the applied thermal load compared to that of the retort treatment was therefore only due to the more rapid and uniform heating performed by OH sterilization, which can be seen in the lower C values.

For PES, on the other hand, there already exists a substantial amount of data on the synergistic inactivation effect of the applied pressure and temperature on spore inactivation (27, 49, 50). This was also acknowledged by the FDA (US Food and Drug Administration) approval for PES treatment, with temperature <121.1°C and pressure 600 MPa (13).

Subsequently, lower C values could be achieved for PES sterilization, through a distinct reduction in temperature and holding time. However, even for samples treated at the same F_0_ values, lower C values resulted for PES treatments compared to those of conventional retort and OH due to rapid and uniform adiabatic heating and cooling. Currently, PES treatment would be feasible for certain niche products that need to be sterilized in-pack and for which a high-quality outcome is the aim, e.g., nutraceuticals as well as baby food. OH is more suitable for applications requiring a high throughput and an out-of-pack treatment, e.g., soups, sauces, fruit preparations, etc. At the present time, to the knowledge of the authors, there are no commercial concepts for in-pack OH treatments.

### Impact of Sterilization Treatments on the Formation of Food Processing Contaminants

Furan and its derivates were analyzed in sterilized carrot–oil model purees and the CRP. Additionally, the treated carrot–oil model purees were also analyzed for the formation of 3-monochloropropane-1,2-diol (3-MCPD) esters. However, due to the low processing temperatures, the treatment types had no influence on the levels of 3-MCPD esters. The detected levels of 3-MCPD esters were significantly below the detection limit of 0.9 μg/kg in all samples. Therefore, the analysis of 3-MCPD esters in the CRP samples were not considered.

Furthermore, the formation of acrylamide was analyzed in CRP samples. Acrylamide levels for all analyzed CRP samples were below the limit of quantification of 30 μg/kg. These findings are in accordance with Commission Regulation (EU) 2017/2158, where benchmark levels of acrylamide in baby food was established to 40 μg/kg (51).

#### Furan and Methylfuran Formation in Model Matrices

Currently, data provided by scientific literature concerning the amounts of furan and its methyl derivates 2-methyl-, 3-methyl-, and 2,5-dimethylfuran (2-MF, 3-MF, and 2,5-DMF) is notably scarce. This is why the European Food Safety Authority (EFSA) has issued a call for the collection of data recording the occurrence of these harmful substances in food (52). It was postulated that exposure to 2-MF, but not 3-MF, would be additive to furan exposure in terms of health consequences from their consumption (53). The formation pathway of methylfurans is connected to the furan pathway since both can have the same precursors (54–56). It is still unknown how the system shifts to either methylfurans or to furan. Maga and Katz (54) postulated that the formation could be dependent upon the precursors.

The content of olive oil used in the model food puree was expected to contain distinct precursors for the formation of furan and its derivates. Other possible contributors to the formation of furan and possible derivates are carotenoids, reducing sugars, as well as proteins from carrots (~0.9 g per 100 g) (57), but also polyunsaturated fatty acids present in the oil used for puree preparation. To the best knowledge of the authors, there is currently no record of studies regarding the formation of methylfurans performed under PES or OH conditions.

As mentioned in *Sterilization Treatments*, the carrot–oil model food puree (Tables 1, 3) was sterilized through PES (including thermal references), as well as through OH and conventional retort, in order to fine tune the analytical method for the quantification of furan and it derivates. The idea of the model food systems is to validate the method and analysis. The system also allows for the creation of a product that is not unlike a real product but specifically contains various potent precursors [e.g., polyunsaturated fatty acids (PUFAs); especially linoleic acid (C18:2) of which rapeseed oil contains ~20% and olive oil ~7% (58); reducing sugar, carotenoids, vitamin C, etc.] to form furan. The formation pathway of furan under thermal conditions is quite well-known, and some publications concerning the influence of PES and thermal treatments on the formation are available (26, 55, 59–64).

**Figure 5:**
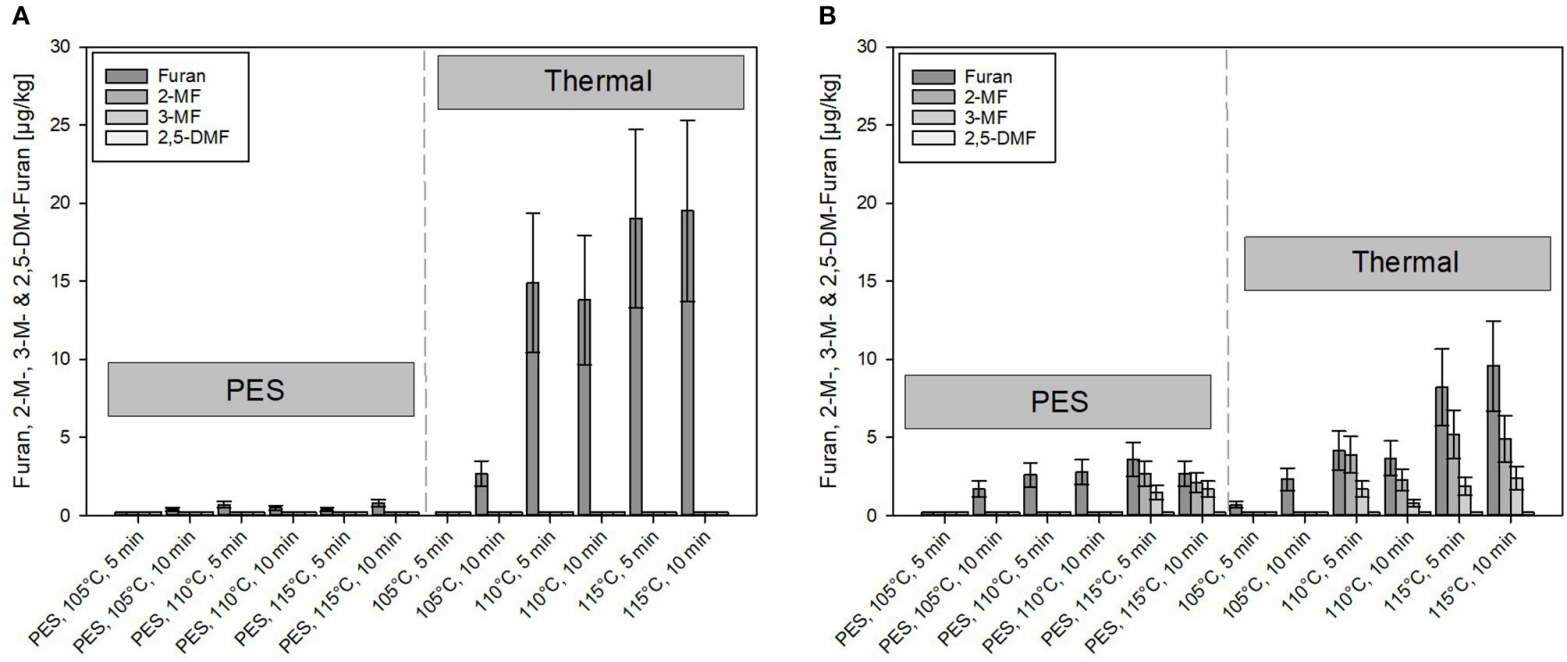
Comparison of furan and its derivates formed under pressure-enhanced sterilization (PES) (600 MPa) and solely thermal conditions. **(A)** Carrot puree with olive oil and **(B)** carrot puree with rapeseed oil. Error bars represent 95% confidence interval.

In the untreated model purees, the content of furan and methylfurans was below the detection limit (0.2 μg/kg). However, furan formation was observed after processing. Figures 5A,B show the formation of furan and its derivates in samples treated by PES (under pressure 600 MPa) and solely heated (thermal reference, no pressure used), with the same temperature time combinations. The heating time of PES and thermal reference samples was controlled to be comparable.

A minimal amount (≤ 0.8 μg/kg) of furan and 2- or 3-MF was formed under PES conditions for the carrot puree containing olive oil, although, to some extent, minor formation occurred for the carrot puree with rapeseed oil (Figure 5B) at 115°C, 5 and 10 min, 600 MPa. Compared to the olive oil, higher levels of furan and its derivates (up to 4 μg/kg) were found in the carrot puree containing rapeseed oil. However, these levels are still quite low compared to published data on occurrence of furan and its derivates in jarred baby food preparations (65).

In comparison, under solely thermal conditions, furan formation starts occurring at 105°C, 10 min under solely thermal conditions. At 110°C, the formation starts to increase with time and temperature. The threshold temperature for the furan formation was reported to be at 110°C (2, 66). Furthermore, the formation of furan under thermal conditions was almost doubled in the system containing olive oil (Figure 5A). Whereas, the formation of 2- and 3-MF did not exist in the aforementioned product. Notably, 2-MF was present in the puree containing rapeseed oil processed at temperatures ≥110°C. This could be attributed to the presence of precursors in the preprocessed oils. Due to the pretreatment of, e.g., rapeseed oils (bleaching, neutralization, deodorization, etc.), where often high temperatures (105°C for bleaching and 270°C for deodorization) are involved, this can lead to lipid oxidation and to the occurrence of, e.g., 2-pentanal and 2-hexanal (67). Adams et al. (67) showed that these compounds can be triggered by amino acids or proteins, which are present in the food system, to react to 2-MF and 2-alkylfuran. The theory was also postulated by Märk et al. (61). This could possibly explain why derivates of furan are formed in the system containing rapeseed oil, rather than furan itself.

The mitigation effect of PES on the formation of furan can be attributed to the lower thermal load applied and the Le Chatelier's principle, which influences the reaction pathway to some extent (26, 64, 68).

**Figure 6:**
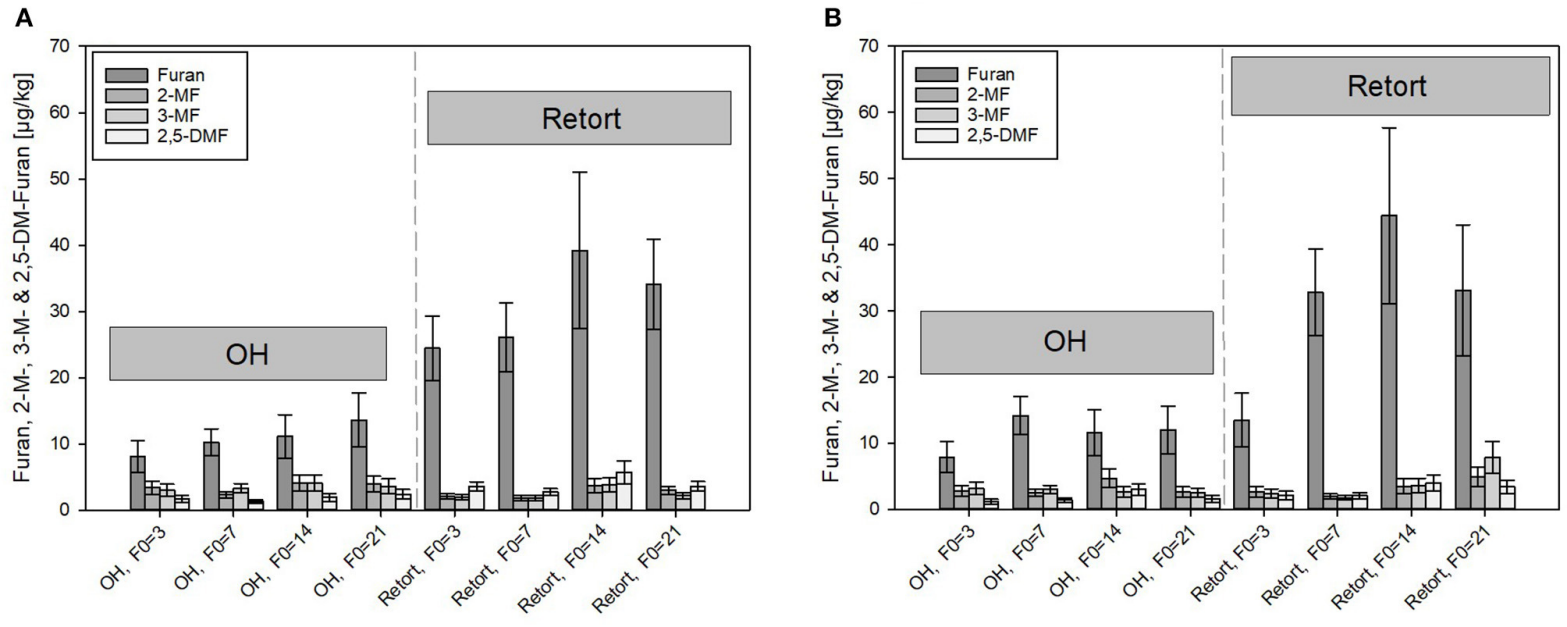
Comparison of furan and its derivates formed under ohmic heating (OH) and conventional retort treatment. **(A)** Carrot puree with olive oil and **(B)** carrot puree with rapeseed oil. Error bars represent 95% confidence interval.

Considerably higher content of furan and its derivates were determined in the carrot-model food systems sterilized by OH and conventional retort (Figure 6). In general, with increasing thermal load, i.e., with higher F_0_ values, higher furan content was found in the samples processed by the retort, whereas lower levels of furan were found in ohmic-sterilized samples than in the retort-sterilized purees. Furan levels in the OH carrot olive oil samples were, however, consistently higher than those found in the PES samples.

After the retorted purees containing rapeseed oil were treated with the highest thermal load of F_0_ = 21 min (33.1 μg/kg), furan formation levels were almost triple those detected after treatments with the lowest thermal load of F_0_ = 3 min (13.5 μg/kg). The levels of furan at F_0_ = 14 min were, surprisingly, slightly higher than those of the samples sterilized with a F_0_ = 21 min. With F_0_ = 14 min, furan levels of 39.2 and 44.4 μg/kg were analyzed in the puree containing olive oil and rapeseed oil, respectively. Differences between the purees containing rapeseed oil and olive oil were within the determination uncertainty. Therefore, the results are not in accordance with furan formation in the thermal PES references done in the lab scale, where an impact of the two different oils was determined. It was assumed that the more severe heat treatments during the retort treatments and the resulting overall higher furan levels overshadowed the impact of the oils. In scientific literature, levels of furan between 6.3 and 85.6 μg/kg were reported in commercially available vegetable-based purees, which is in general accordance with the results obtained in this study (65). In addition, the reported level of furan in strained carrots (intended as baby food) with 27.8 μg/kg by Becalski et al. (53) is in alignment with the obtained results.

Under OH conditions, distinctly lower furan and 2- or 3-MF was formed as compared to those found in retort conditions. Furan found in the carrot–oil purees sterilized by OH ranged from 7.9 μg/kg (F_0_ = 3 min) to a maximum of 13.6 μg/kg (F_0_ = 21 min), a reduction of 64% at F_0_ = 3 min, and a reduction of 41% at F_0_ = 21 min as compared to the retort samples. The higher relative reduction rate at lower F_0_ values is in accordance with the determined C values and due to the lower holding times at temperatures above 110°C, which was reported to be the threshold temperature for furan formation (66). In the OH samples, the furan content increased only slightly with higher F_0_ values. The observations are therefore in good accordance with results from Hradecky et al. (30), who also reported significantly lower furan levels in carrot-based purees sterilized by OH as compared to those of conventional retort sterilization. The authors also reported only a minor impact of the sterilization value on the furan formation for the OH treatment. The reduced furan formation was linked to the uniform and rapid heating nature of the OH treatment, which allows a reduction in the overall thermal load. However, although care has been taken, the sample handling could have an impact on the furan content in the OH samples. After cooling inside the OH chamber, purees were transferred as rapidly as possible to small sample containers (100 ml) and immediately stored at −30°C. A partial evaporation of furan and its derivates during the transfer, due to their volatile nature, cannot be excluded. In order to avoid volatilization effects, in-pack OH sterilization treatments need to be conducted to further investigate the impact of OH on the furan formation as opposed to furan formation in the conventional retort. Similar observations were reported when the furan formation for ultrahigh temperature (UHT, continuous heating)-treated foods was investigated. Compared to retorted samples, lower furan formation, and almost no increasing furan levels with increasing F_0_ values were observed (69). This is probably linked to faster heating rates of an open system application.

In general, lower levels of the furan derivates (2- and 3-MF and 2,5-DMF) compared to furan were formed in all treated purees. The concentrations of the derivates in the retorted samples were in the range of 2.1–7.9 μg/kg. Again, the thermal load affected the formation of the compounds. At F_0_ = 21 min, distinctly higher levels of all three derivates were detected as compared to F_0_ = 3 min. Similar to furan, the formation of the methylfurans was not affected by the presence of the two different oil types.

#### Furan and Methylfuran Formation in CRP

**Figure 7:**
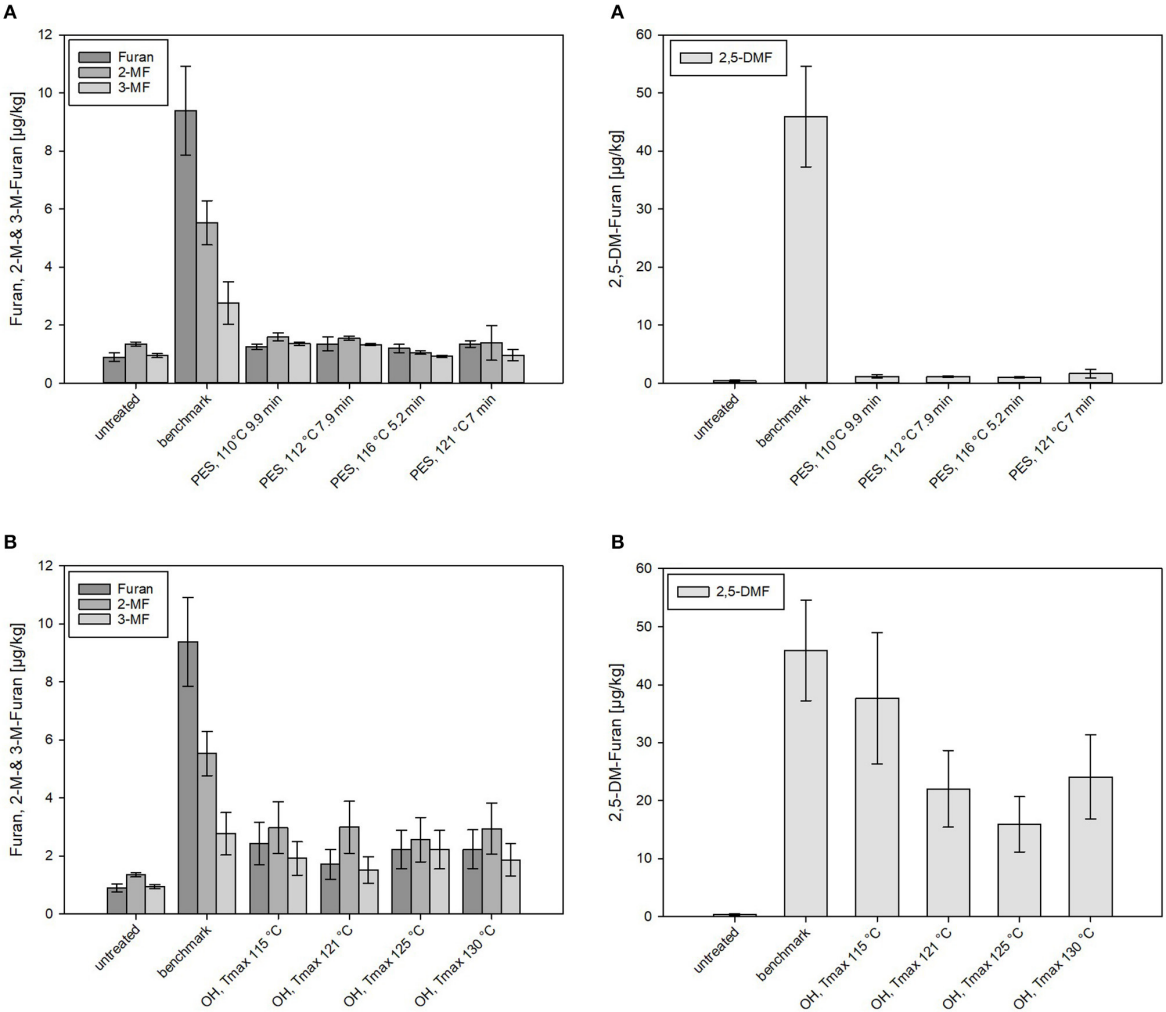
Levels of furan and its derivates formed in chicken rice puree (CRP) after **(A)** pressure-enhanced sterilization (PES) and **(B)** ohmic heating (OH) treatments as well as industrial benchmark sterilization in comparison to the untreated sample. OH and benchmark samples were sterilized at a target F_0_ = 7 min. OH samples were treated by different maximum temperatures (115, 121, 125, and 130°C). For 130°C OH treatment, the F_0_ value was 13.2 min. Error bars represent 95% confidence interval.

In the CRP samples, lower overall furan levels were found as compared to the carrot–oil model purees. One possible reason for this could be the higher water content and therefore lower concentration of precursors (carotenoids, carbohydrates, proteins, etc.) available in the CRP. Interestingly, significantly higher 2,5-DMF levels were found in the OH-treated and industrial benchmark purees, and 2,5-DMF was also the dominant process contaminant identified in CRP. The results of the furan formation (including its derivates) in the PES- and OH-treated samples are shown in Figures 7A,B, respectively. Analyzed OH samples (with different maximum temperatures) and the industrial sterilized sample (benchmark) equaled a F_0_ = 7 min (except OH T_max_ = 130°C that equaled to F_0_ = 13.2 min). All PES treatments theoretically lead to a *B. amyloliquefaciens* inactivation of 12 log_10_.

Nominal amounts (≤ 1.5 μg/kg) of furan and derivates were found in PES samples even under sterilization conditions equal to the industrial benchmark (121°C, 7 min). Distinctly higher amounts of furan and especially 2,5-DMF were determined in thermally (benchmark and OH) treated products. Levels between 2 and 3 μg/kg of furan, 2-MF, and 3-MF were detected in the OH-sterilized samples, significantly higher levels than occurred in the PES samples but significantly lower levels than occurred in the benchmark samples (9.4 ± 1.5 μg/kg) (Figure 7). In the benchmark samples, 2-MF (5.5 ± 0.8 μg/kg) and 3-MF (2.8 ± 0.7 μg/kg) levels were significantly lower than the formed furan. In the puree examined, the main contributors to the formation of furan and its derivates were, based on the recipe of CRP, probably carrots (carotenoids and sugars) and chicken meat (proteins/amino acids). The presence of chicken meat, however, in the recipe did not lead to a particularly high furan formation as compared to the carrot–oil model purees. This is not in accordance with results of other authors who reported enhanced furan formations in starch-based gels by the addition of proteins (70). On the other hand, the results are in accordance with data reported by Hradecky et al. (30), who found lower levels of furan in vegetable purees containing chicken meat (13–14 μg/kg for F_0_ = 8–16 min) than in samples without chicken (29–32 μg/kg for F_0_ = 11–20 min).

**Figure 8:**
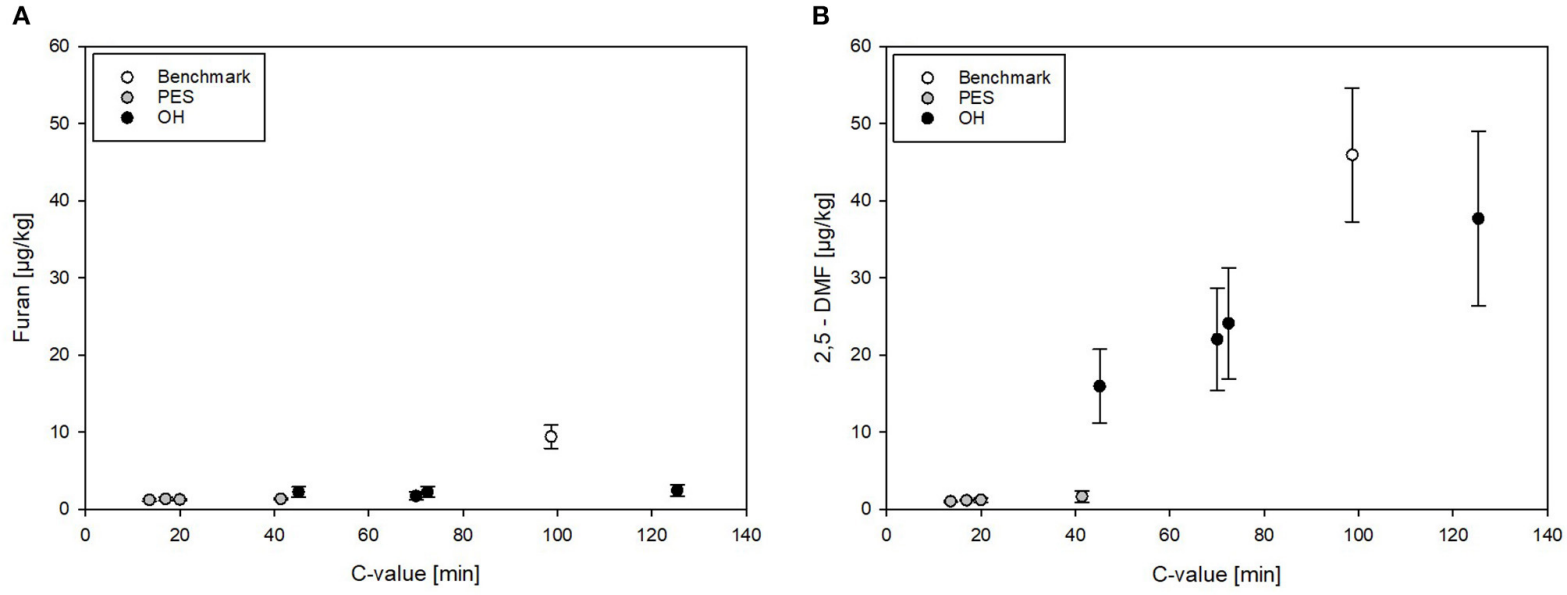
**(A)** Furan and **(B)** 2,5-DMF levels in chicken rice puree (CRP) plotted against the C value resulting from pressure-enhanced sterilization (PES), ohmic heating (OH), and industrial benchmark treatments. Error bars represent 95% confidence interval.

To be able to compare the different sterilization technologies (PES, OH, and benchmark retort) in relation to the applied thermal load, furan levels formed in treated CRP were plotted against the determined C values for all treatments, illustrating graphically the mitigation potential of the different technologies. In CRP, the furan levels were overall relatively low. The levels of furan in PES- and OH-sterilized samples (independent from the attained C value) were below 2.5 μg/kg. The furan level in the industrial benchmark samples was only mediocre although significantly higher than all PES and OH samples, including the one OH sample with a higher C value (Figure 8A).

Overall, relatively high amounts of 2,5-DMF were formed in all thermal (OH and benchmark) samples but not in PES-sterilized samples (Figures 7, 8B). 2,5-DMF is a recently found new derivate of furan and not much is known about its occurrence and formation (3). According to the results, applied pressure had an influence on the formation pathway, since by addition of pressure, lower amounts were formed in comparison to the amounts formed in the thermal processes. The highest level of 2,5-DMF was found in the industrial benchmark puree (45.9 μg/kg). An impact of the T_max_ was observed in the samples sterilized by OH: the minimum amount of 2,5-DMF having been formed with a T_max_ of 125°C (15.9 μg/kg) and the maximum amount with a T_max_ of 115°C (37.7 μg/kg). These observations were in accordance with the C values determined for the purees (Table 5). Interestingly, neither in the untreated CRP (<3 μg/kg) nor in the sterilized carrot–oil model purees (<5 μg/kg at up to F_0_ = 21) were such high 2,5-DMF levels found.

When comparing the 2,5-DMF formation in PES, OH, and benchmark samples in relation to the C values, the high mitigation potential of PES can be seen (Figure 8B). Even at a C value comparable to the lowest C value reached by OH, significantly lower 2,5-DMF levels were found. In the OH samples, the formation of 2,5-DMF increased with increasing C value, with the highest level at T_max_ = 115°C (C value = 125.4 min). It can be clearly seen that, due to its potential to reduce the C value by more efficient heating, OH has a higher potential for mitigation of furan and its derivates than the retort process.

It can be concluded that pressure had an influence on the formation pathway of furan and its derivates. Lower amounts of furan and its derivates were formed when the parameter pressure was added than under the same conditions in the thermal process. In this case, PES offers a double benefit by reducing toxicological potential as well as by improving the microbiological safety of the food. In the future, more samples and analytes need to be tested to fully understand the influence of pressure on the reaction pathway. OH offers the advantage of more rapid and uniform heating and, therefore, the potential to reduce thermal damage during sterilization treatments. The analyses showed that the applied analytical method was able to detect even low amounts of furan.

In general, knowledge of methylfuran formation in food is very scarce and not well understood (3). For 2-MF, a pathway based on a Maillard-type reaction, including reducing sugars and specific amino acids, was proposed in model food systems (55, 61). 2-MF was reported to be formed primarily when amino acids were present. The formation was stated to be based on aldol reactions of Strecker aldehydes of certain amino acids, with lactaldehyde (from threonine) being one of the key intermediate products (55). Furthermore, also unsaturated aldehydes originating from oxidation of unsaturated fatty acids, like linolenic acid, might be relevant precursors for the corresponding methylfurans (3). However, because there is still no data to be found dealing specifically with the formation of 2,5-DMF, further research is necessary.

Very little data are available concerning the presence of 2,5-DMF in food products. Shen et al. (71) analyzed various food products for the occurrence of furans and methylfurans including 2,5-DMF. They found levels of 0.5–41 μg/kg of 2,5-DMF in various soy sauce, vinegar, and coffee products, but the levels were always lower than furan and 2-MF. The highest amount of 2,5-DMF was found in a milk sample with 88.8 μg/kg. However, in fruit- and vegetable-based baby food purees, no 2,5-DMF was detected. Therefore, compared to other vegetable-based baby food preparations, the levels of 2,5-DMF found in the present study (up to 45.9 μg/kg) in a carrot-based puree were surprisingly high.

In commercially available flour- or cereal-based baby food meals, Habibi et al. (72) found comparably high levels of 2,5-DMF (69.4–230.3 μg/kg). Furthermore, in five out of six samples, 2,5-DMF was the dominant process contaminant, with higher levels than furan and 2-MF. Thus, according to scientific literature, cereal- and milk-related preparations were found to be more prone to 2,5-DMF formation. The CRP investigated in the current study contained a cereal source (10% rice), which might have contributed to the 2,5-DMF formation. However, more research is necessary to investigate the formation pathway and to identify the precursors of the furan derivate.

### Impact of the Sterilization Treatments on the Carotenoid Content

**Figure 9:**
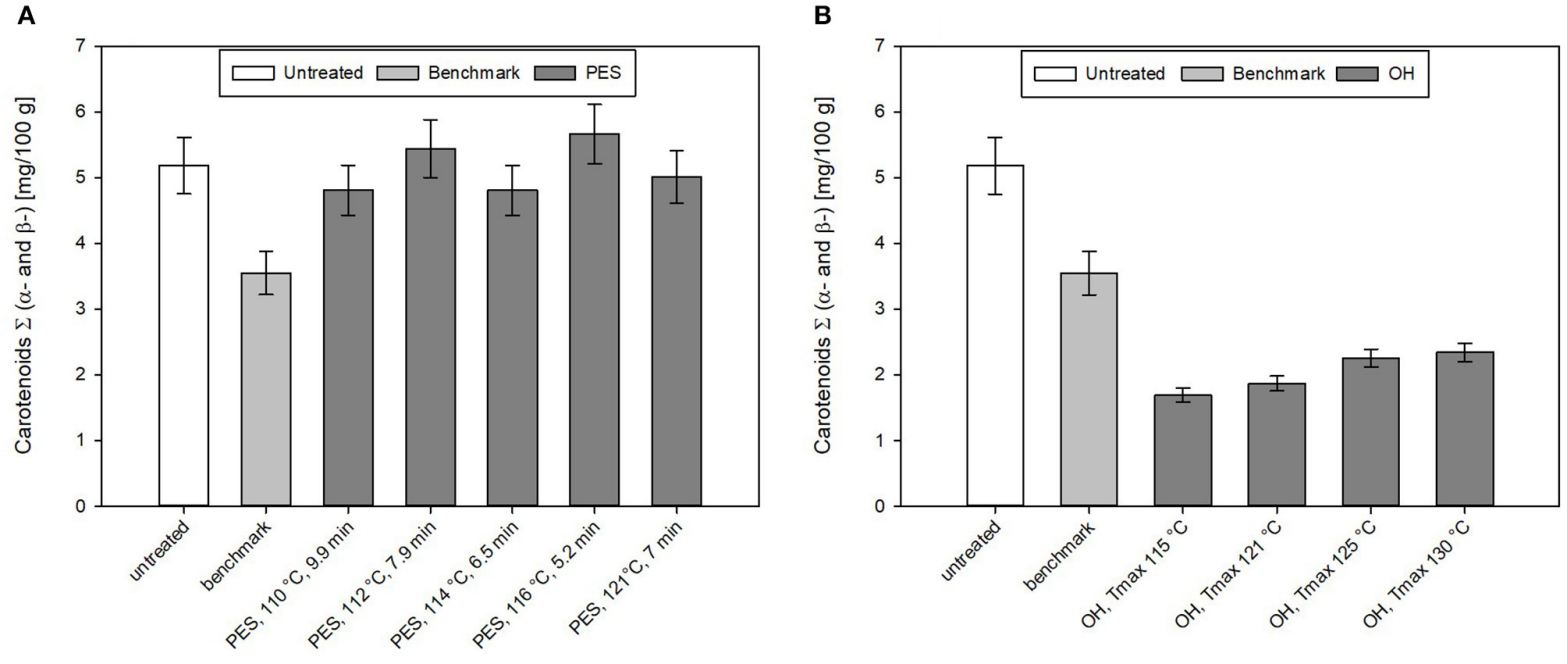
Carotenoid content of chicken rice puree (CRP) after the **(A)** pressure-enhanced sterilization (PES) and **(B)** ohmic heating (OH) treatment as well as industrial benchmark sterilization in comparison to the untreated sample. OH and benchmark samples were sterilized at F_0_ = 7. OH samples were treated by different maximum temperatures (115, 121, 125, and 130°C). For 130°C OH treatment, the F_0_ value was 13.2 min. Error bars represent 95% confidence interval.

Carotenoids are a typical quality parameter often affected by thermal treatments, and therefore, were analyzed in the CRP before and after the treatments. The results of the carotenoid content of all CRP samples can be found in Figure 9.

For the untreated sample, an average total carotenoid content (including α- and β-carotene) of 5.2 ± 0.4 mg/100 g was determined. The recipe contained 40% carrots, and if one uses the average amount of carotenoids found in carrots with 12.3 mg/100 g [based on AUSNAT (73)] to roughly estimate the amount of carotenoids in the sample, ~4.9 mg/100 g should be found in the untreated sample. This is in accord with the amount of carotenoids found in the untreated sample. In general, for different PES treatments, not only minor losses with ~7% but also increases of the carotenoid content up to ~9% can be observed. The industrial benchmark process leads to a loss of 31% of the original carotenoid content.

By OH, no better retention of the analyzed total carotenoids (α- and β-carotenoids) compared to the industrial benchmark samples was observed. All sterilization treatments resulted in a reduction in the total carotenoid levels in the purees (Figure 9A). In the purees sterilized by OH, carotenoid levels ranged between 4 and 5 mg/100 g; this corresponded to a loss of 50–60% based on the original carotenoid content.

Literature data on the influence of PES on carotenoids are scarce. Sánchez et al. (74) investigated the carotenoid content in six different vegetables (carrot, red pepper, tomato, broccoli, spinach, and green pepper) and their changes under PES conditions (625 MPa, 5 min, 117°C) and found similar results directly after the treatment. The raw carrots contained in a total of α- and β-carotene an amount of 13.86 mg/100 g. This is similar to what was found in the Australian food database (73). The results indicated that after preheating, the carotenoids increased by 15%. Sánchez et al. (74) mentioned that the rise could be explained by the fact that the application of pressure and/or temperature lead to a softening of the plant tissue and denaturation of proteins that could help to release carotenoids. After the PES treatment (625 MPa, 5 min, 117°C), they accounted loss of ~7% of the initial carotenoid content. This was in accordance to loss (4–6%) of carotenoids in the CRP, treated at 117 and 118°C, although in this case, no comparison was performed with a thermal process, respectively. In terms of carotenoid retention directly after the treatment, PES seems to be superior to the thermal benchmark processing. Future studies should be conducted to evaluate how storage may influence the carotenoid profile for PES and for a thermal benchmark process. Here, especially for PES, packaging and its barrier properties will play an important role.

From previous literature, it is known that thermal treatments may lead to better accessibility and bioavailability of carotenoids. However, severe thermal treatments may reduce the carotenoid content in food (75). The available data on this topic is therefore contradictory. Dhuique-Mayer et al. (76) investigated the impact of different thermal treatments on the carotenoid content of sweet-potato-containing baby purees. Sterilization treatments (123°C, 30 min, F_0_ = 16.1 min) lead to a loss of 32% of all-trans-β-carotene in the puree. Similar results were reported for orange pepper puree after an intense heat treatment (130°C, 1 h), lutein was reduced by about 90% and zeaxanthin by about 50% (77). In carrot juice, loss of 60 and 55% for α- and β-carotene were observed during retort sterilization (121°C, 30 min) (78). Milder sterilization treatments (110°C, 5 min) were reported to not significantly reduce the amount of carotenoids in carrot juice (79).

Very little data are available regarding the effect of OH on the carotenoid content of vegetable juices or purees. Yildiz et al. (46) investigated the β-carotene content of spinach puree during OH treatment up to a temperature of 90°C. Slightly higher β-carotene concentrations were observed after OH treatment than by water bath heating. However, overall, only a minor impact of the two treatments on β-carotene was reported. Additionally, the voltage gradient (10–40 V/cm) did not have a significant influence on the β-carotene concentration (46). An OH treatment and a conventional water bath heating of carrot cubes up to 97°C resulted in higher β-carotene concentrations than that of raw carrots. The results were linked to textural degradation and, therefore, a better extractability of the carrot tissue. No distinct impact of the OH treatment compared with that of the conventional thermal treatment was identified (80). On the other hand, Mannozzi et al. (81) demonstrated that during OH of carrot juice at temperatures above 40°C, the amount of carotenoids decreased due to the presence of oxygen.

When comparing the two volumetric sterilization technologies, only PES was able to improve the carotenoid retention as compared to that of the industrial benchmark process. By OH, carotenoid levels were slightly lower than those of the benchmark sample. This was unexpected, as the more rapid and uniform OH treatments also led to lower final C values than those of the industrial benchmark sample. However, it cannot be ruled out that the sample handling during the OH treatments might have influenced the carotenoid degradation, although the sample was cooled inside the treatment chamber and quickly transferred into the sample container and immediately frozen. The additional exposure to oxygen during the transfer from the treatment chamber to the sample container might have affected the carotenoid content.

### Color

Color is an important quality characteristic of fruit- and vegetable-based products and a major factor affecting sensory perception and consumer acceptance of foods. It can be assumed that the color of the food for PES as well as for OH is closer to the untreated product since shorter processing time and/or, in general, a lower thermal load is applied in comparison to traditional retort conditions. This results in the preservation of heat-labile bioactive compounds (e.g., chlorophyll, carotenoids, anthocyanins, etc.) responsible for the color of fruits and vegetables.

**Figure 10:**
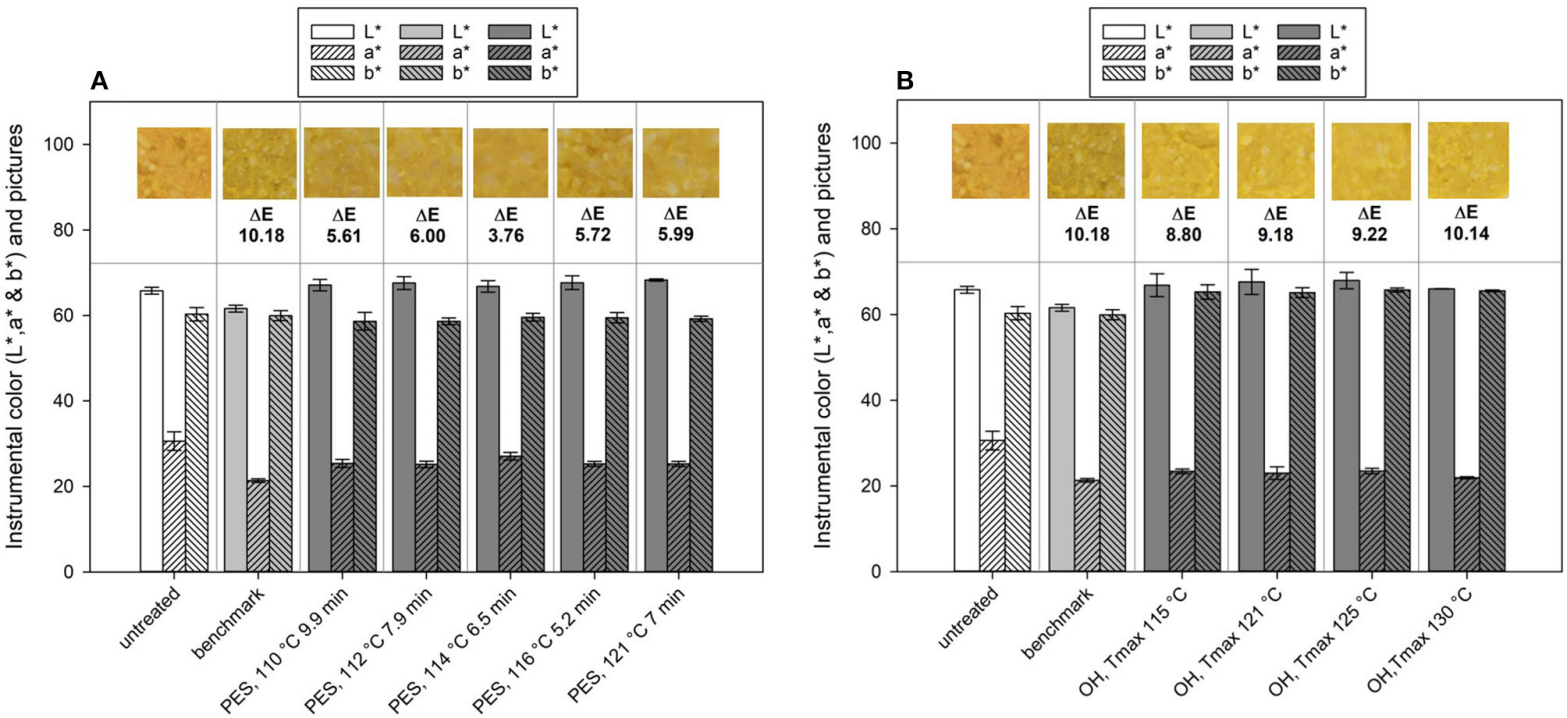
Lab color values and calculated ΔE values of chicken rice puree (CRP) before and after processing and their representative pictures of the **(A)** pressure-enhanced sterilization (PES) and **(B)** ohmic heating (OH) treated samples as well as thermal benchmark sample.

The changes in color (L^*^, a^*^, b^*^ values, and ΔE) of the PES- and OH-treated samples are shown in Figures 10A,B, respectively. All the tested parameters for OH equal to F_0_ = 7 min (except OH T_max_ = 130°C equaled F_0_ = 13.2 min) and for PES theoretically lead to the inactivation of 12 log_10_. The L^*^, a^*^, and b^*^ measurements were conducted with the same system to have a better overall comparison of the tested technologies.

If one looks at the changes in the single values of L^*^, a^*^, and b^*^, changes between the untreated and the industrial benchmark process occurred especially for the L^*^ and a^*^ value, whereas b^*^ remained almost the same (Figure 10). After the benchmark treatment, the sample was slightly darker (lower L^*^ value) and lost some of the redness (lower a^*^ value). The PES samples mainly showed changes in the a^*^ (lower) and the b^*^ value (lower), meaning a loss in red and yellow, respectively, orange. The ΔE of the industrial benchmark process was the highest with ~10.18 in comparison to that of the PES-treated samples, which ranged between 3.76 and 6.00. Therefore, PES had a smaller impact on the color in comparison to the industrial benchmark, which, in this case, can be attributed to the lower thermal load applied (C value, Table 5). The selected treatment conditions under pressure lead to a theoretical 12 log_10_ inactivation and only affected the color of the final product to a minor degree. The calculated C values for these PES treatments ranged from 13 to 19 min and lead to similar color changes in terms of ΔE. Even the sample with the highest thermal load (121°C, 7 min, 600 MPa) and with a C value of 41 min had a similar ΔE as the other samples. This may indicate that a maximum color change for this product, based on the ΔE, was reached within the tested temperature–time–pressure domain. The literature concerning the influence of PES on color is scarce. There is one publication from Al-Ghamdi et al. (82), which looked at the influence of pressure-assisted thermal sterilization (PATS, with initial temperature 98°C, mean temperature over 5 min of treatment at 600 MPa ~107.5°C) on nutrients and quality of pumpkin, butternut squash, pea, beetroot, and purple potato purees. Since 121.1°C was not attained during the process portrayed in the publication, and due to the given temperature profile, their process should rather be understood as a pressure-enhanced process. All treatments induced color changes and led to ΔE values in the range from 3 to 14, contrary to the untreated samples. Especially, the greenish and purplish color of peas and the purple potato puree changed extremely under PATS. Pumpkin (orange), butternut squash (yellow), and beetroot (dark red) were not affected as much. A comparison with a thermal benchmark process was not part of the study and would have been helpful to evaluate and classify the degree of change induced by the high-pressure high-temperature process.

The OH treatment also significantly changed the color of the untreated puree (Figure 10B). In general, a shift from orange in the untreated samples to yellow in the sterilized samples was observed. Determined ΔE values of the OH treatments were in a similar range, between 8.80 and 10.14. Therefore, the OH samples had only slightly lower ΔE values than the industrial benchmark samples. However, differences between the benchmark sample and the OH samples can be identified when looking closely at the L^*^, a^*^, and b^*^ values and the photos. The industrial benchmark sample was slightly darker (lower L^*^ value) than were the OH samples, and the yellowness was slightly lower (lower b^*^ value). Only a slight impact of the varying T_max_ values could be observed within the OH samples, the highest T_max_ (130°C) possessing the highest ΔE value (10.14). The C values of the OH treatment did not directly affect the color retention. The difference between the OH treatments and the benchmark sample might be due to the overprocessing of the outer areas in the glass jar during the industrial retort sterilization (see Figure 4), leading to even higher C values in those areas.

For carrot juice, a shift from orange to yellow was reported under intense thermal treatments (78). This is in accordance with the observations made in this study, as carrots were the main component responsible for the color in the puree.

A few authors have studied the impact of OH treatments on color retention of fruit and vegetable purees. Yildiz et al. (46) reported good color retention of spinach purees for water-bath-heated and ohmically heated samples up to 90°C. However, the authors also observed a slightly higher deviation from the untreated samples and a higher browning effect with OH. In contrast to those results, an enhanced color retention by OH blanching compared to water bath blanching in peach puree was observed. By a more rapid and more uniform heating, the targeted enzyme inactivation could be reached more rapidly, and therefore, the color retention could be improved (47).

While comparing the two innovative technologies, OH had a greater influence on color change than PES and had a similar ΔE as the thermal retorting. Interestingly, the changes induced by the technologies differed slightly, whereas for OH, the a^*^ value was reduced and the b^*^ value increased. For PES, both a^*^ and b^*^ value decreased. No changes were observed for either technology on the L^*^ value. Nevertheless, the color change for OH and PES is inclined toward a brighter color, as the brownish darker color corresponded to the industrial benchmark process in comparison to the untreated sample. This could be attributed to the fact that the degree of nonenzymatic browning reaction is lower for both OH and PES due to less cooking damage as a result of volumetric heating and shorter processing time.

### Texture

To the best knowledge of the authors, no comparison has been conducted to date of the influence of three different technologies, including two emerging technologies and the conventional retorting on the textural behavior, based on a textural profile analyses of a selected food.

**Figure 11:**
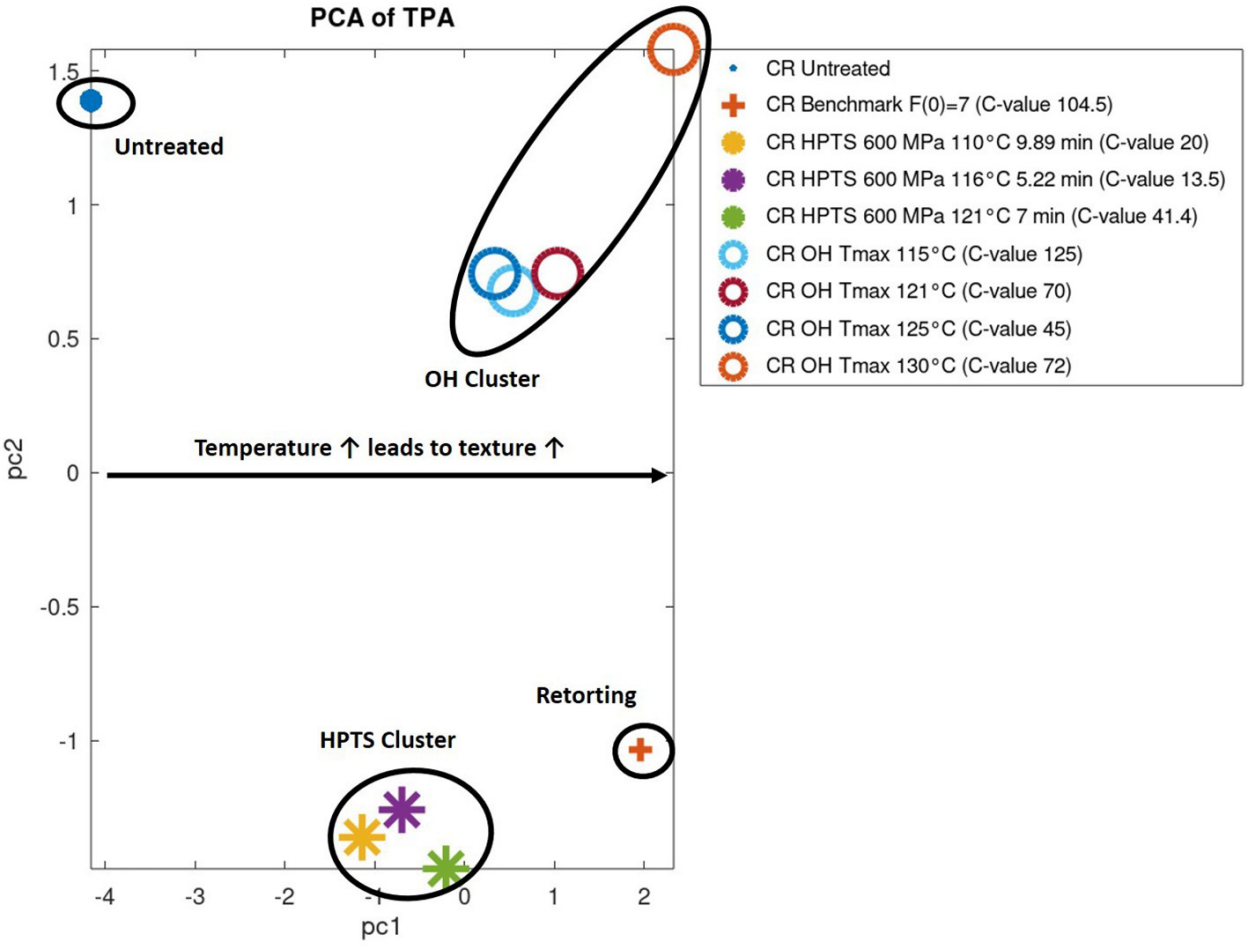
Principle component analysis (PCA) of textural profile analysis (TPA) results of chicken rice puree (CRP) samples sterilized by pressure-enhanced sterilization (PES), ohmic heating (OH), and benchmark retort and the untreated sample.

The texture profile analyses (TPAs) were carried out as already described under *Texture*. Based on the results of the TPA, a principle component analysis (PCA) was performed to find possible clusters in the textural behavior of the foods, depending on their treatment. Further, all data were normalized (put into relation to the untreated sample) prior to applying the PCA. The results of the PCA based on the results obtained by TPA from the PES, OH, and the industrial benchmark can be found in Figure 11. PC 1 contained the majority (80%) of the information and can be used as an orientation for interpreting the results.

Clusters occurred for PES-treated, OH-treated, untreated, and sterilized benchmark samples. The main attributes contributing to the clustering were firmness and stickiness of the treated product. The PES-treated samples increased in firmness and stickiness in correlation to the treatment temperature in comparison to the untreated sample from 50 to 100%, respectively, 33–80%. Whereas the OH-treated samples had a similar increase in firmness (247–260%) as the benchmark process (255%), depending on the treatment, some variations occurred, e.g., the OH sample treated at T_max_ = 130°C (C value of 72 min, right-hand corner) showed a severe increase of 452% in firmness. The stickiness was ~60% higher in comparison to the industrial benchmark process, but only minor changes occurred in the different parameter settings. Overall, in terms of change in texture, compared with the untreated (but precooked) sample, the PES samples are closer in value to the untreated sample as are the ohmically or the benchmark samples.

This was probably the effect of a lower thermal load applied to the product by OH and PES, which, at least for some samples, is indicated by the C value. Otherwise, the final target temperature also seems to be important. Therefore, the gelling and texturing effect is not as pronounced as for the industrial benchmark. Nevertheless, a tendency concerning the textural fingerprint of different technologies could be shown for the first time for these emerging technologies in comparison to the benchmark retorting and an untreated reference sample. More research and data need to be evaluated to fully understand the influence of the different technologies on texture.

## Conclusion

The present study shows that the two emerging technologies OH and PES are promising sterilization technologies for vegetable purees. The thermal load applied to the product, of both technologies, could be reduced compared to the conventional retort treatment, without neglecting food safety and quality. Compared to the conventional retort, lower C values could be reached by OH due to a more rapid and uniform heating of the puree samples. Even lower C values resulted from the PES treatments due to the synergistic inactivation effect of temperature and high pressure and the possibility to reduce processing temperature. This resulted in a better retention of color, bioactive compounds such as carotenoids, and texture, as well as a reduced formation of food processing contaminants in samples processed by the innovative sterilization technologies. Particularly, a significant reduction in the formation of both furan and its derivates could be observed, compared to the retorted samples. In the sterilized CRP samples, 2,5-DMF was found to be the dominant process contaminant, displaying higher values than furan, 2-MF, and 3-MF. High levels of 2,5-DMF were formed in thermally (industrial benchmark retort and OH) sterilized CRP samples but not in PES-treated purees. Hence, PES was identified to be the most promising technology for the mitigation of the formation of furan and its derivates.

To lower the thermal impact, the best processing conditions for ohmically heated CRP were found for higher maximum temperatures, in this case 125°C. For PES, 600 MPa, 116°C held for 5.22 min was found to be the most promising parameter in terms of all quality and safety parameters tested. Additionally, trials with the selected process parameters (Table 5) were done with 200 g of CRP to evaluate the scalability of the PES treatment. Similar results for the physiochemical characteristics and formation of FPCs were observed compared to the small-scale trials. Results were, however, not included in the manuscript due to confidentiality issues. Further research is needed to validate these findings, which should include storage tests and sensorial evaluations. Overall, these results will help to understand the potential of the tested emerging technologies, leading to improvements in practical implementation.

## Data Availability Statement

The original contributions presented in the study are included in the article/Supplementary Material, further inquiries can be directed to the corresponding author/s.

## Author Contributions

All authors listed have made a substantial, direct and intellectual contribution to the work, and approved it for publication.

## Conflict of Interest

The authors declare that the research was conducted in the absence of any commercial or financial relationships that could be construed as a potential conflict of interest.
