# Supplementary material for: Gentle Sterilization of Carrot-Based Purees by High-Pressure Thermal Sterilization and Ohmic Heating and Influence on Food Processing Contaminants and Quality Attributes
Source: Front Nutr. 2021 Mar 18;8:643837. doi: 10.3389/fnut.2021.643837 (PMC8020890; doi:10.3389/fnut.2021.643837)
Supplement: Supplementary file 1 [file Data_Sheet_1.DOCX]

**Supplementary Material**

S1 Supplementary Materials and Methods

S1.1 Spore strains, spore preparation and inoculation

Using a method described elsewhere (Paidhungat et al., 2002) for *Bacillus amyloliquefaciens* (Technische Mikrobilogie Weihenstephan, 2.479, Fad 82), sporulation was induced at 25, 30 and 35 °C. The different sporulation temperatures were selected to alter the resistance of the spores to the high-pressure high-temperature treatment. It is known that the lower the sporulation temperature, the more resistant the spore becomes to the PES treatment (Cortezzo and Setlow, 2005; Michiels and Bartlett, 2008; Olivier et al., 2012; Raso et al., 1998). Based on the carried-out inactivation kinetics, the most resistant spore batch was selected. In the first step, a stock solution with vegetative cells was made from a frozen spore culture. For this purpose, 3 mL of the frozen spore suspension were dissolved in 50 mL sterile nutrient solution (Nutrient Broth, CM001, Oxoid Ltd., Basingstoke, England), to which 0.5 mL (corresponds to 10 µL/mL) streptomycin was dissolved and placed in an incubator. The solution was shaked continuously at 37 °C for at least 24 h until a severe turbidity became visible.

The sporulation was carried out on 2xSG agar plates (according to Leighton & Doi, 1971). The harvest was carried out when 90% of the spores were phase bright under the light microscope, which took 4–5 days. The spore suspension was cleaned by repeated centrifugation (3- fold at 5000 g), washed with cold distilled water (4 °C), and was treated with sonication for 1 min. The cleaned spore suspensions contained ≥95% phase bright spores and nearly no spore agglomerates, as was verified by a particle analysis system (FPIA 3000, Malvern Instruments, Worcestershire, U.K.).

The final spore concentration was 10^10^ CFU/mL. The spore suspensions were stored in the dark at 4 °C until further use.

For the high-pressure high-temperature treatment, depending on the wanted inoculation level, the matrix, either N-(2-acetamido)-2-aminoethanesulfonic acid (ACES) buffer solution (pH 7, 0.05 M) or puree, was mixed with needed amount of spore suspension and vortexed for 20 s. The inoculated sample was then placed in 1 mL micro-reaction tubes (Nunc Cryo Tybe Vials ™, Thermo Fisher Scientific, Waltham, Massachusetts, United States) and stored on ice until treatment.

**Table 6: Empirical values for the start of high-pressure treatment of ACES buffer. T_oil = temperature of the oil bath, T_ch = temperature in the sample chamber, T_st = temperature at the start of the pressure build-up, T_end = target treatment temperature of the sample.**

| T_oil [°C] | T_ch [°C] | T_st [°C] | T_end [°C] |
| --- | --- | --- | --- |
| 110 | 108 | 65 | 105 |
| 115 | 113 | 70 | 110 |
| 120 | 119 | 75 | 115 |
| 125 | 124 | 80 | 121 |

For the thermal treatment class capillary method, described elsewhere (Mathys, 2008) was used. All temperature treatments for the ACES-buffer as well as the puree were carried out in glass capillaries. For this purpose, 100 µL of the spore suspension was mixed in a reaction tube with 900 µL matrix and vortexed. The suspensions were filled into thin glass capillaries with an internal diameter of 1 mm, an external diameter of 1.3 mm, and a length of 100 mm (Kleinfeld Labortechnik GmbH, Gehrden, Germany). Prior using the capillaries, each glass capillary was first heated in the middle under the Bunsen burner and bent into a V-shape. The spore suspension was then injected into the glass capillary using a sterile syringe (diameter 0.9 mm, Sterican, B. Braun Melsungen AG, Melsungen, Germany) heat sealed and stored on ice until use.

The thermal treatment in glass capillaries was conducted with the same temperatures (105, 110 and 115 °C) at 0.1 MPa. A thermostatic bath (cc2, Huber GmbH, Offenburg, Germany) filled with silicon oil (M40.165.10, Huber GmbH) was used for the treatment. Following thermal treatment, the samples were immediately transferred into an ice bath.

After the high-pressure and thermal treatment a serial dilution with Ringer-solution down to 10^-7^ was performed and survivors after the two treatments were measured by plate count in two replicates using nutrient agar. Colonies of *Bacillus amyloliquefaciens* were incubated at 37 °C and were counted after 2 days.

S1.2 Analysis of spore inactivation and regression analysis

To calculate the isokinetic lines, the individual inactivation kinetics at the respective temperature were first adjusted using the Weibull function Eq. 1 $\log\left( \frac{N}{N_{0}} \right)=-{(\frac{t}{k})}^{b}$, t = treatment time [min], *k* = inactivation rate and *b* = shape parameter.

Prior to the calculation of the isokinetic line, each inactivation (e.g., PES CRP 105–115 °C) was approximated with a Weibull fit. Shape parameter *b* and inactivation rate *k* were recorded, and *a* mean *b* calculated. Based on the mean *b*, the inactivation kinetics were re-fitted and *k* determined. *k* in dependency of the temperature was calculated based on an exponential fit. Now it is possible to solve $\log\left( \frac{N}{N_{0}} \right)=-{(\frac{t}{k})}^{b_{mean}}$ since $k\left( T \right)=a*e^{-c*T}$ and can be expressed for either a 6 or a 12 log_10_ inactivation $t\left( T \right)=6^{\left( \frac{1}{b_{mean}} \right)*a*e^{-c*T}}$. To find a possible process window, all solutions that solve this function need to be found. To make this all a little easier and faster, a calculation routine was programmed in Octave. The standardized method “Microbiology of food and animal feeding stuffs. Control of stability of preserved and assimilated products, Routine method” NF V 08-408 (AFNOR, 1997) was used to evaluate if the treatment conditions were suitable to produce a shelf-stable low-acid product by PES. Therefore, 2 samples of each treatment condition were stored for 21 days at room temperature and 37 °C. The norm also includes one storage test at 55 °C for 7 days, but instead it was chosen to store the samples for 21 days although the norm only requires 7 days. After the storage time had ended, the following chart was used to determine if the product is stable or not ( Figure 12).


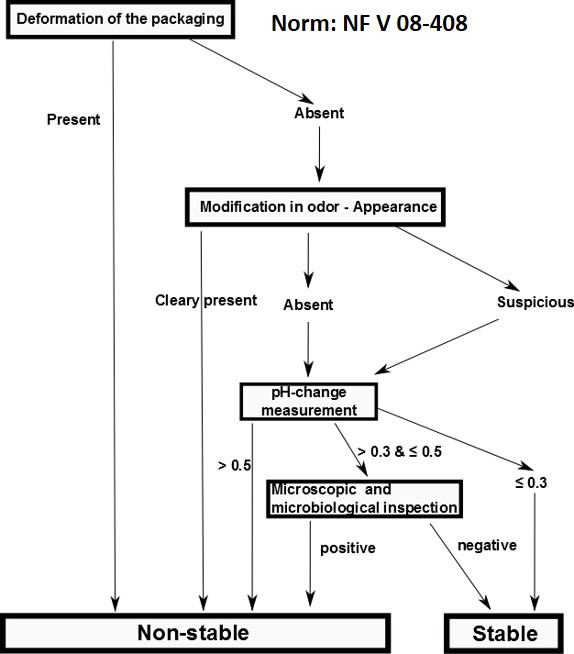


**Figure 12: Chart to interpret the results of the storage trials conducted at room temperature and 37 °C. Adapted from Standard NF V 08 408.**

This includes a pH measurement of the samples stored at room temperature (reference) and 37 °C, depending on the pH difference between the same samples differently stored, further investigations are necessary. After the storage period, microbiological analyses on nutrient agar were also conducted to evaluate if barro/thermo resistant microorganisms survived the treatment in the samples. To sum up, this means that a product is considered stable if the samples show: No deformation of the container, no modification of the appearance and smell, no pH difference >0.5 unit between the samples, and no specific microflora in the different samples under the microscope. All trials were carried out in duplicates.

S2. Supplementary results and discussion

S2.1 Bacterial spore inactivation PES

The PES results were used to determine the needed inactivation kinetics based on which the isokinetic lines were calculated. The reached inactivation level was always a 6 log_10_ reduction, which was used to extrapolate an 8, 9 and 12 log_10_ reduction.

i) Determination of the most resistant *Bacillus amyloliquefaciens* due to different sporulation temperatures (25, 30 and 35 °C)

As mentioned by other authors and research groups, if spores are sporulated at lower temperatures, they become more resistant towards high-pressure and high-temperature conditions (Cortezzo and Setlow, 2005; Michiels and Bartlett, 2008; Olivier et al., 2012; Raso et al., 1998). On the other hand, spores become more sensitive towards temperature. Therefore, it is suggested that for the selection of PES resistant spores, reduced sporulation temperatures or the addition of minerals to the sporulation media should be adopted to get as close as possible to the worst-case scenario. The current opinion in the literature is that sporulation temperatures to some extent influence the inner spore membrane physiology and its corresponding systems and mechanisms (Olivier et al., 2012). As mentioned by Knorr *et al.* and Reineke *et al.* (2011; 2013) the target structure of high-pressure in spores is the inner spore membrane, with dipicolinic acid (DPA)-channels and germinant receptors. Coretzzo and Stelow (2005) mentioned that with decreasing sporulation temperature the fluidity and permeability of the inner spore membrane increases. This is a similar mechanism reported for vegetative cells where grown at higher temperature or with different minerals, the fluidity of the phospholipid double layer increases as well and the shift from fluid to gel under high-pressure conditions is retarded and therefore, the resistance is increased (Casadei et al., 2002; Molina-Höppner et al., 2013). Currently, there is no proof of this mechanism, but the data found in the literature suggest such a mechanism.

Figure 13 (A & B) shows the inactivation results based on isokinetic lines for a 6 log_10_, 8 log_10_ and 12 log_10_ inactivation for different sporulation temperatures of *Bacillus amyloliquefaciens* spores inoculated in a real food system (vegetable-meat puree) and ACES-buffer under thermal and PES conditions. Some of the selected temperature-time combinations for the CRP can be found in Table 7.

**Table 7: Possible process windows for the CRP for different sporulation temperatures and inactivation levels under PES conditions. BA = *Bacillus amyloliquefaciens.***

|  | BA 35 CRP (-6 log_10_) | | BA 25 CRP (-6 log_10_) | | BA 25 CRP (-8 log_10_) | | BA 25 CRP (-12 log_10_) | |
| --- | --- | --- | --- | --- | --- | --- | --- | --- |
| **600 MPa** | **T [°C]** | **t [min]** | **T [°C]** | **t [min]** | **T [°C]** | **t [min]** | **T [°C]** | **t [min]** |
| **1** | **104.00** | **7.16** | **104.00** | **16.33** | **107.24** | **15.47** | **110.12** | **9.89** |
| **2** | **106.04** | **5.85** | **106.00** | **11.88** | **109.22** | **11.36** | **112.16** | **7.94** |
| **3** | **108.08** | **4.77** | **107.00** | **10.23** | **111.20** | **8.34** | **114.20** | **6.50** |
| **4** | **110.12** | **3.89** | **108.00** | **8.64** | **113.18** | **6.13** | **116.07** | **5.22** |
| **5** | **112.16** | **3.18** | **110.00** | **6.40** | **115.16** | **4.50** | **118.11** | **4.20** |
| **6** | **114.20** | **2.59** | **111.00** | **5.41** | **117.14** | **3.30** | **120.15** | **3.83** |
| **7** | **116.07** | **2.15** | **112.00** | **4.66** | **119.12** | **2.42** | **-** | **-** |
| **8** | **118.11** | **1.76** | **114.00** | **3.39** | **120.20** | **2.05** | **-** | **-** |
| **9** | **120.15** | **1.43** | **116.00** | **2.51** | **122.00** | **1.55** | **-** | **-** |

The isokinetic lines indicate that for lower sporulation temperatures lead to an increased resistance, e.g., in ACES buffer with spores sporulated at 35 °C a 6 log_10_ inactivation takes at 600 MPa, 108 °C ~5 min and at 25 °C the same inactivation takes 7.5 min at 108 °C. Furthermore, the thermo-baro protective effect of the food matrix in comparison to the buffer systems is also visible. Lower sporulation temperatures make spores more resistant towards high-pressure and high-temperature conditions, but more sensitive towards solely temperature processing.


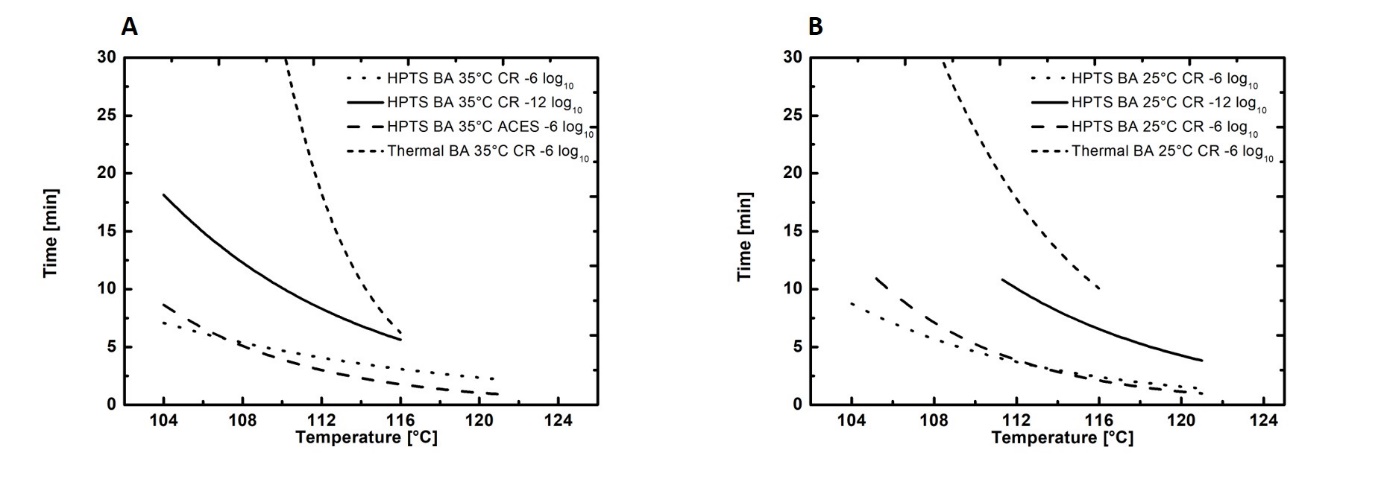


**Figure 13: Isokinetic lines for a -6 & -12 log_10_ inactivation in CRP and ACES-Buffer under PES and thermal conditions. A) *Bacillus amyloliquefaciens* spores sporulated at 35 °C, B) *Bacillus amyloliquefaciens* spores sporulated at 25 °C**

These findings are in accordance with those made by Margosch *et. al* (2004) that *Bacillus amyloliquefaciens* is the most resistant and comparable to the resistance of the pathogenic spore former *Clostridium botulinum* if sporulated at 30 °C. Due to the higher resistance of *Bacillus amyloliquefaciens* sporulated at 30 °C this batch of spores was chosen to conduct the rest of experiments.

ii) Validation of the calculated isokinetic line and storage trials

In order to verify the calculated data, individual temperature-time combinations of the isokinetic line of 6 log_10_ were examined for their actual inactivation (Table 7). To do so, 9 temperature time combinations were selected, and the samples were inoculated with 10^8^ spores/mL (detection limit 7.5 log_10_) of *Bacillus* *amyloliquefaciens* sporulated at 25 °C and then treated at the selected conditions (Figure 14). The reached inactivation, for the selected temperature time combinations (black dots), fluctuates between -5.99 log_10_ and -6.49 log_10_ units and therefore validates the model based on the single inactivation kinetics performed for the CRP at 105,110 and 115 °C at 600 MPa. The same was done for 8 log_10_, here an inoculum of 10^10^ in the ratio of 1:10 was used to get to 10^9^ spores/mL (detection limit 8.5 log_10_), with also 9 temperature time combinations (Table 7). The isokinetic line, the parameters tested, and the corresponding inactivation can be found in the upper part of Figure 14. The reached inactivation, for the selected temperature-time combinations (black dots), fluctuates between -7.71 log_10_ and -8.49 log_10_ units, and therefore also validates the model based on the single inactivation kinetics performed for the CRP at 105,110 and 115 °C at 600 MPa.


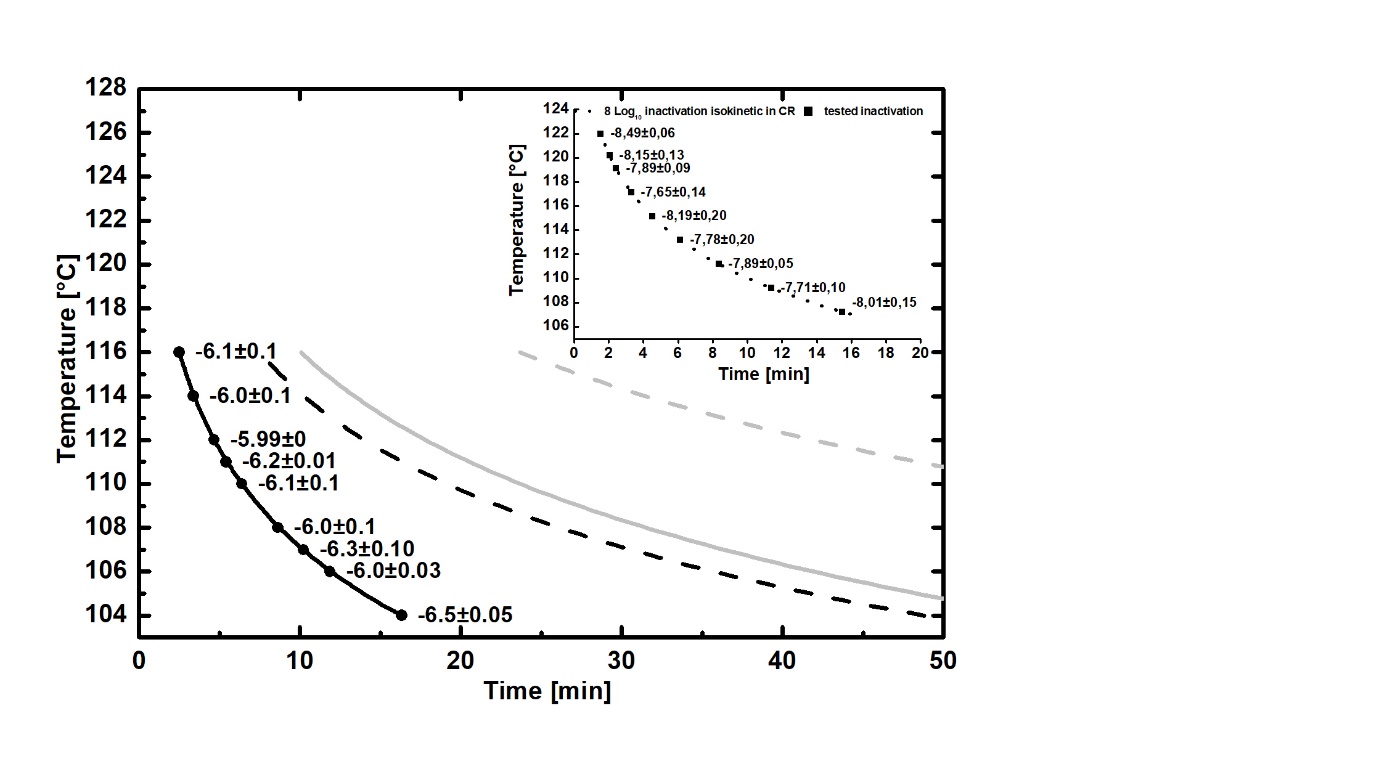


**Figure 14: Validation of the model *via* inactivation studies for a calculated 6 log_10_ and 8 log_10_ inactivation of *Bacillus* *amyloliquefaciens* in CRP under PES conditions. Black solid line 6 log_10_ and black dashed line 12 log_10_ inactivation under PES conditions; gray solid line 6 log_10_ and gray dashed line 12 log_10_ inactivation under thermal conditions. Black dots indicate the tested conditions as well as the achieved inactivation level. Upper right corner: Isokinetic line (black dotted line) for 8 log_10_ inactivation. Black squares indicate the tested conditions as well as the achieved inactivation level.**

According to current practice, a 12 D-concept ergo, an inactivation leading to a reduction of 10^12^ CFU/mL of spores, is regarded as microbial safe. Therefore, an extrapolated -12 log_10_ isokinetic line (dashed black line) is shown in Figure 14. Like the 12-D concept, this cannot be done through laboratory experiments and cannot be demonstrated since it is impossible to have such high bacterial spore load in food without changing its specific properties. In addition, the isokinetic lines for a -6 log_10_ and a -12 log_10_ inactivation for a thermal treatment were also calculated. Here, the time effect and the synergistic effect of pressure and temperature on the inactivation of spores for PES become obvious (Olivier et al., 2015). All temperature-time combinations of the -12 log_10_ isokinetic line for PES are below the -6 log_10_ isokinetic of thermal treatment. For a safe thermal sterilization, it takes at least 25 minutes at 115 °C. Through the validation of the -6 log_10_ and -8 log_10_ isokinetic line, it can be assumed that the CRP treated with different temperature-time combinations for a -12 log_10_ inactivation at 600 MPa, is also sterile. Considering the implementation in the industry, the holding time of the PES process should not be longer than 10 minutes (

Figure 14 & Table 8). Therefore, the optimized processing of the product by PES is a case-by-case consideration. To have an idea of the thermal load applied to the product and to have better comparability with other technologies, the C-value concept was chosen. The C-value, other than the F-value (lethality effect during the treatment) looks at the degradation of sensorial and nutritional attributes during the treatment. The C-value can be calculated by integrating the temperature-time curve of the applied process.

**Table 8: Selected C-values for a 12 log_10_ inactivation, based on the treatment conditions.**

| Parameters | C-value |
| --- | --- |
| PES 110 °C, 9.89 min | 19.95 |
| PES 112 °C, 7.9 min | 16.92 |
| PES 116 °C, 5.22 min | 13.51 |
| PES 121 °C, 7 min | 41.38 |
| Thermal 121 °C, 7 min | 65.30 |

Table 8 shows some of the selected C-values for a 12 log_10_ inactivation. Here it is obvious that the thermal load applied to the product, depending on the process (except for the F_0_ = 7 under PES), is lower by 69–79% as for the thermal benchmark process. Even under the same “sterilization conditions” at 121°C the PES process has a thermal load that is 36% lower in comparison to the thermal benchmark process. The selected parameters from Table 8 are the basis of all other trials conducted.

To further validate the correctness of the model for the 12 log_10_ inactivation, the sets of parameters from Table 7 (BA 25 CRP -12 log_10_) were used to apply an “indirect” challenge study. Therefore, the samples were inoculated with 10^9^ spores/g and treated with the conditions shown in Figure 15, Figure 16 and Table 7 for a 12 log_10_ inactivation. By inoculating the samples with 10^9^ spores/mL, the food properties were not altered since a 1:100 dilution was used to achieve this spore level, and further this closet one could get to the 12-D concept in realistic terms. The standardized method “Microbiology of food and animal feeding stuffs: Control of the stability of preserved and assimilated products, Routine method” NF V 08-408 (AFNOR,1997) was used to evaluate if the treatment conditions were suitable to produce a shelf-stable low-acid product by PES (Figure 12).


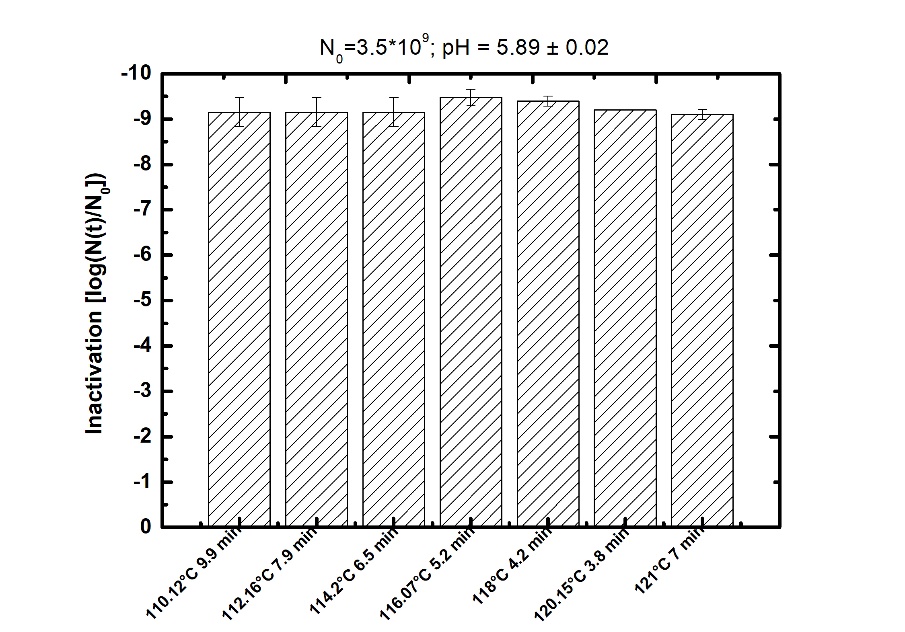


**Figure 15: Results of the challenge study directly after the treatment at 600 MPa.**

Directly after the treatment, as already shown in Figure 15 all the inoculated spores were inactivated. The total spore load in the sample was 3.5*10^9^ CFU/g of *Bacillus amyloliquefaciens* spores and the initial pH value of the sample was 5.89 ± 0.02.

As mentioned in the norm, the samples need to be checked for possible survivors, respectively growth as well as pH changes after 21 days stored at room temperature and 37 °C. The results of this study are shown in Figure 16.

**
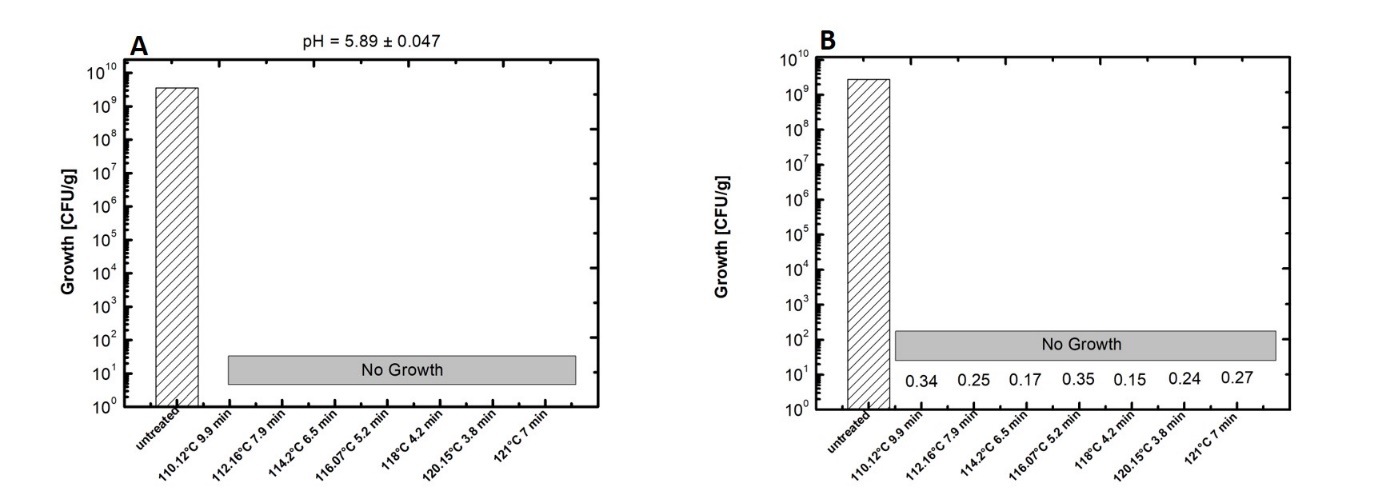
**

**Figure 16: Results of the challenge study based on NF V 08 408. A) Samples stored at room temperature for 21 days and B) Samples stored at 37 °C for 21 days.**

For the samples stored at room temperature (Figure 16 A), no growth was detected for all samples treated at PES conditions and no significant pH change were observed. The untreated sample still contained the number of spores that were inoculated at day 0. For the samples stored at 37 °C (Figure 16 B), the pH change for some samples were in the range of <0.3 & ≤0.5 and therefore, the microbiological growth was also checked *via* plate count. As it can be derived from the graph also here, no growth occurred for all tested samples except for the untreated sample. The challenge study showed that the calculated temperature and time combinations at 600 MPa based on the calculated model are feasible to not only obtain a safe product directly after the treatment, but also after storage. To the knowledge of the authors, the only research article that used a similar approach was Sevenich *et al.* (2015), which determined the effect of PES on furan and microbiological safety at lab and pilot scale level in different food systems (vegetable puree, sardine in olive oil, tuna in brine etc.). The results there also indicate that temperatures ≥110 °C are needed to obtain a sterile and safe product. To validate and to make further statements on the “real” shelf-life of the products, additional studies need to be considered, such as degradation kinetics of valuable compounds (vitamins, etc.) and rise of unwanted compounds (lipid oxidization, etc.) during storage, actual real time storage tests for the desired shelf-life and sensorial evaluation. Nevertheless, the conducted “challenge” study gives a first impression if the used technology offers microbial safety and takes the first hurdle.

The model seems to work for the tested product and this is why for the scale-up for a possible scale-up, the following parameter combinations for a 12 log_10_ inactivation could be tested: 110 °C, 9.89 minutes, 600 MPa; 116 °C, 5.22 min, 600 MPa; 121 °C, 600 MPa, 7 minutes (as a direct comparison to the benchmark process used in the industry with an F_0_ = 7).
