# Supplementary figures and images for: Gentle Sterilization of Carrot-Based Purees by High-Pressure Thermal Sterilization and Ohmic Heating and Influence on Food Processing Contaminants and Quality Attributes
Source: Front Nutr. 2021 Mar 18;8:643837. doi: 10.3389/fnut.2021.643837 (PMC8020890; doi:10.3389/fnut.2021.643837)

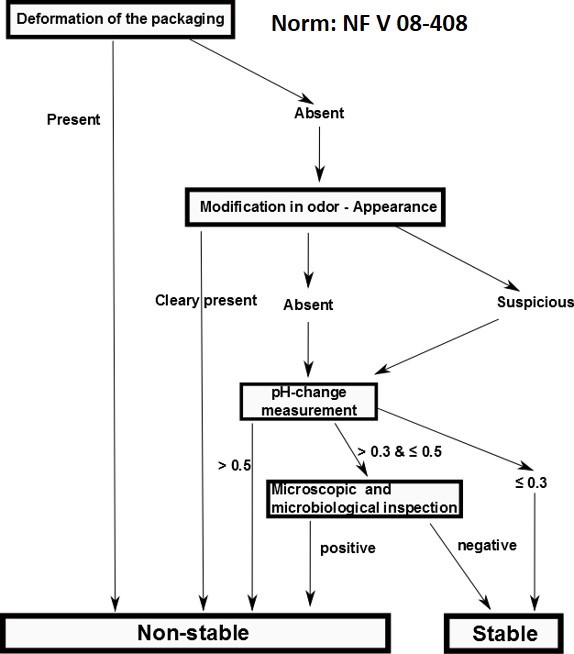

Supplement: Supplementary file 2 [file Image_1.JPEG]

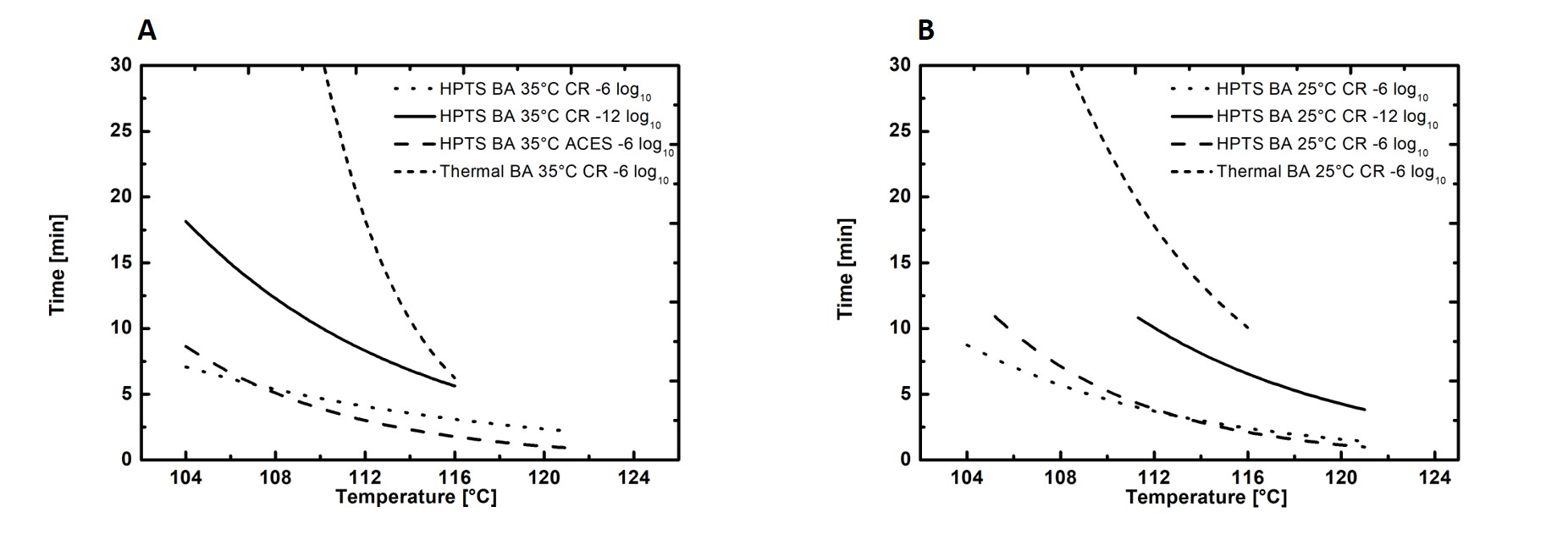

Supplement: Supplementary file 3 [file Image_2.JPEG]

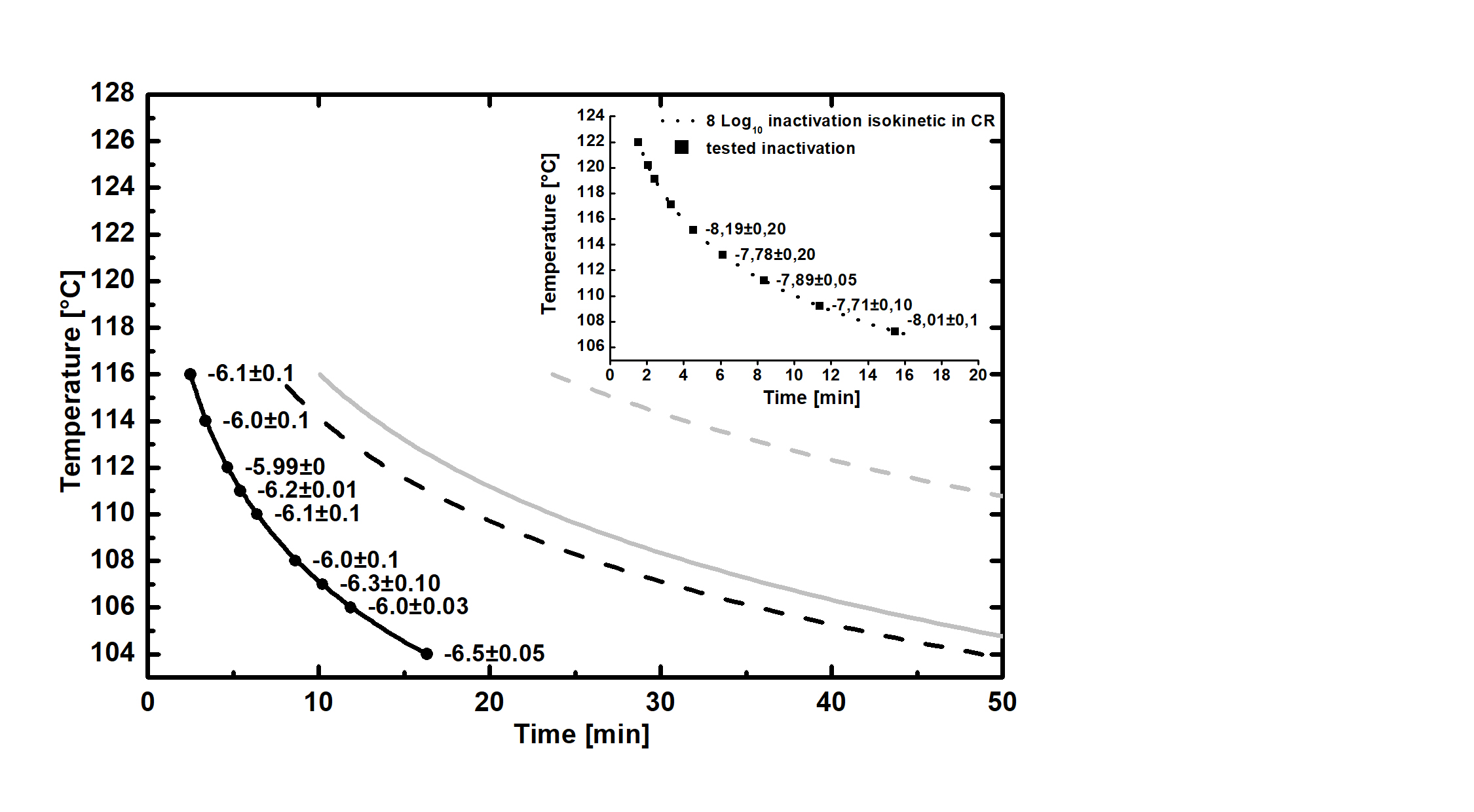

Supplement: Supplementary file 4 [file Image_3.JPEG]

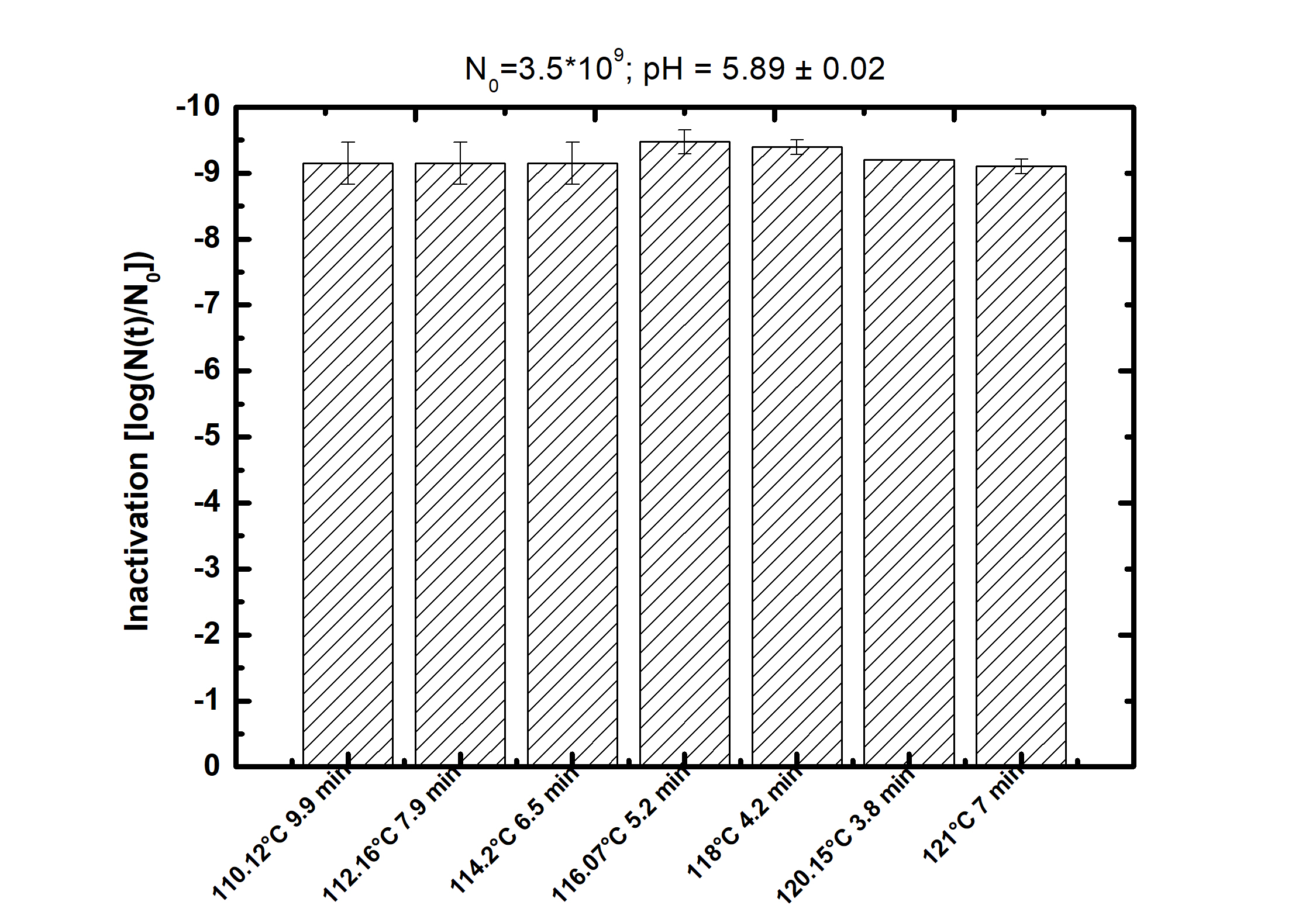

Supplement: Supplementary file 5 [file Image_4.JPEG]

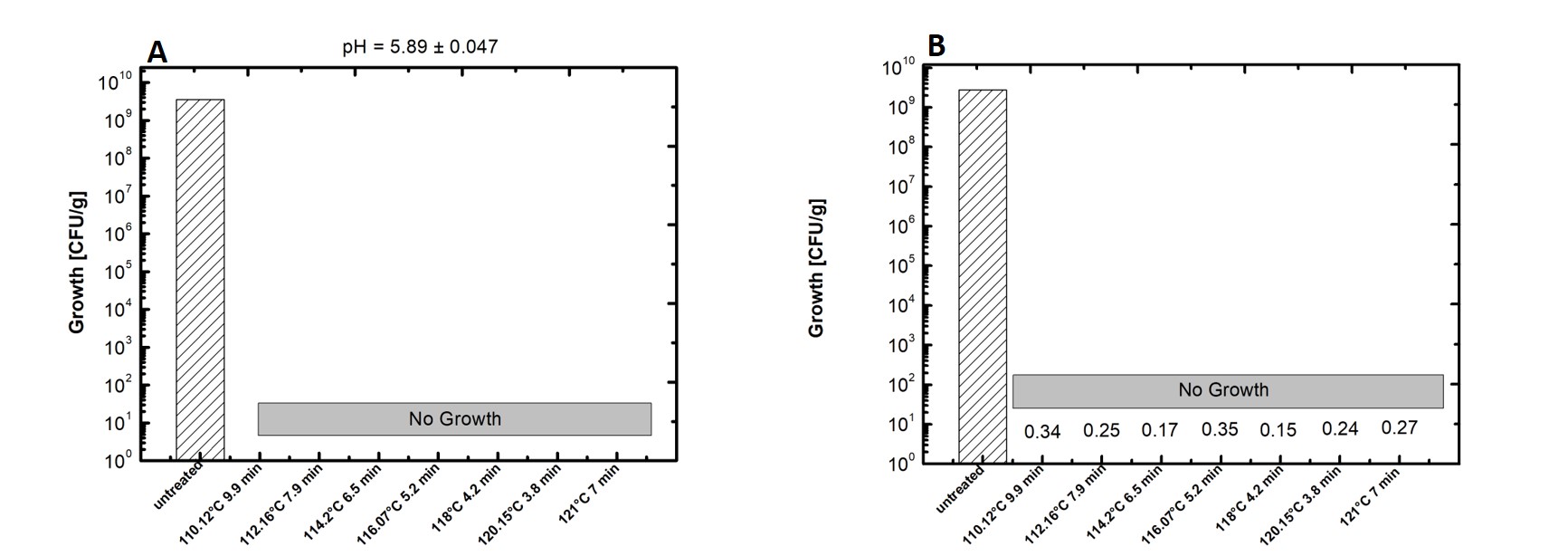

Supplement: Supplementary file 6 [file Image_5.JPEG]
